# Understanding structure–properties relationships of porphyrin linked to graphene oxide through π–π-stacking or covalent amide bonds

**DOI:** 10.1038/s41598-022-16931-8

**Published:** 2022-08-04

**Authors:** Anna Lewandowska-Andralojc, Ewelina Gacka, Tomasz Pedzinski, Gotard Burdzinski, Aleksandra Lindner, Jessica M. O’Brien, Mathias O. Senge, Aleksandra Siklitskaya, Adam Kubas, Bronislaw Marciniak, Justyna Walkowiak-Kulikowska

**Affiliations:** 1 Faculty of Chemistry, Adam Mickiewicz University, Uniwersytetu Poznanskiego 8, 61-614 Poznan, Poland; 2 Center for Advanced Technology, Adam Mickiewicz University, Uniwersytetu Poznanskiego 10, 61-614 Poznan, Poland; 3 Faculty of Physics, Adam Mickiewicz University, Uniwersytetu Poznanskiego 2, 61-614 Poznan, Poland; 4 Helmholtz-Zentrum Dresden-Rossendorf, Institute of Ion Beam Physics and Materials Research, Bautzner Landstraße 400, 01328 Dresden, Germany; 5 School of Chemistry, Chair of Organic Chemistry, Trinity Biomedical Sciences Institute, Trinity College Dublin, The University of Dublin, 152-160 Pearse Street, Dublin 2, Ireland; 6 Institute for Advanced Study (TUM-IAS), Focus Group-Molecular and Interfacial Engineering of Organic Nanosystems, Technical University of Munich, 85748 Garching, Germany; 7 Institute of Physical Chemistry, Polish Academy of Sciences, Kasprzaka 44/52, 01-224 Warsaw, Poland

**Keywords:** Graphene, Photochemistry

## Abstract

Two graphene oxide nanoassemblies using 5-(4-(aminophenyl)-10,15,20-triphenylporphyrin (TPPNH_2_) were fabricated by two synthetic methods: covalent (GO-CONHTPP) and noncovalent bonding. GO-CONHTPP was achieved through amide formation at the periphery of GO sheets and the hybrid material was fully characterized by FTIR, XPS, Raman spectroscopy, and SEM. Spectroscopic measurements together with theoretical calculations demonstrated that assembling TPPNH_2_ on the GO surface in DMF-H_2_O (1:2, v/v) via non-covalent interactions causes changes in the absorption spectra of porphyrin, as well as efficient quenching of its emission. Interestingly, covalent binding to GO does not affect notably neither the porphyrin absorption nor its fluorescence. Theoretical calculations indicates that close proximity and π–π-stacking of the porphyrin molecule with the GO sheet is possible only for the non-covalent functionalization. Femtosecond pump–probe experiments revealed that only the non-covalent assembly of TPPNH_2_ and GO enhances the efficiency of the photoinduced electron transfer from porphyrin to GO. In contrast to the non-covalent hybrid, the covalent GO-CONHTPP material can generate singlet oxygen with quantum yields efficiency (ΦΔ = 0.20) comparable to that of free TPPNH_2_ (ΦΔ = 0.26), indicating the possible use of covalent hybrid materials in photodynamic/photothermal therapy. The spectroscopic studies combined with detailed quantum-chemical analysis provide invaluable information that can guide the fabrication of hybrid materials with desired properties for specific applications.

## Introduction

Among graphene derivatives, graphene oxide (GO) is particularly interesting due to functional groups capable of anchoring functional molecules through non-covalent/covalent interactions. One strategy to fabricate hybrid materials is to functionalize graphene with photochemically active molecules. Among dyes, porphyrins are well suited to construct graphene-based composites due to their excellent photoactive properties^1^. Photoinduced electron transfer (PET) ability has prompted porphyrins to be widely used as photosensitizers in artificial photosynthetic devices^1–5^. In addition, porphyrins are known for long-lived triplet states that enhance the formation of singlet oxygen through energy transfer, and this feature is also critical to improve the efficiency of photodynamic treatments^6–8^.

Considering the above, it is timely and important to prepare hybrid materials consisting of graphene and photoactive units such as porphyrins and study their behavior in the context of application in solar energy conversion or photodynamic therapy. The conversion of light energy to energy-rich molecules (solar fuel) through photocatalytic processes starts with the photoinduced generation of energy-rich electrons. Therefore, the PET process in those hybrid materials plays a key role in converting solar light into chemical energy or electricity. The occurrence of PET in hybrid materials consisting of porphyrins and graphene materials has been demonstrated for several systems^9–12^, and efficient PET has also been shown to enhance photocatalytic hydrogen production^13,14^.

In addition to solar energy conversion, an association of different graphene oxides with photosensitizers has allowed the development of multifunctional photodynamic/photothermal platforms for tumor ablation and metastasis prevention^15–18^. The use of graphene oxides in PDT (photodynamic therapy) is more challenging since the electron acceptor capability of graphene counteracts the generation of ROS (reactive oxygen species). Disabling this alternative deactivation channel is mandatory to assure good ROS production.

Elucidating the factors that affect the efficiency of PET and singlet oxygen generation in porphyrin/graphene materials is crucial for knowledge-driven the design of nanomaterials and their use in solar-energy conversion or PDT.

One of the most critical factors influencing the interaction between porphyrin and graphene material is the synthesis protocol for the composites. Generally, the synthesis of dye/graphene oxide composites requires either covalent or non-covalent functionalization^19–21^. The non-covalent strategy relies on fundamental molecular interactions such as π–π-stacking, hydrogen bonding, or charge attraction between graphene and organic molecules. Covalent functionalization reactions such as the formation of amide or ester linkages are more complex; however, the resulting composite materials are more stable.

Surprisingly, despite these recent advances in the field of porphyrin/graphene nanoassemblies, there are no reports comparing covalent and non-covalent functionalization of graphene oxide with the same porphyrin. Therefore, in the present contribution, we fabricated two GO nanoassemblies using 5-(4-(aminophenyl)-10,15,20-triphenylporphyrin (TPPNH_2_) using two different synthetic methods: covalent (GO-CONHTPP) and noncovalent (GO-TPPNH_2_) bonding. The main objective of the current study was to elucidate whether the type of functionalization covalent vs. non-covalent is privileged for photoinduced electron transfer or singlet oxygen generation. Time-resolved spectroscopic measurements revealed that only the non-covalent assembly of TPPNH_2_ and GO enhances the efficiency of the photoinduced electron transfer from the porphyrin to GO. In contrast to the non-covalent hybrid, the covalent GO-CONHTPP material can generate singlet oxygen with comparable yields to free TPPNH_2_, indicating the possible use of covalent hybrid materials in photodynamic/photothermal therapy. The results clearly demonstrate that the type of functionalization: covalent vs. non-covalent affects the hybrid properties and thus their potential application.

## Experimental

### Reagents and materials

Graphene oxide **(**GO-powder < 35 mesh. C/O atomic ratio = 2.5–2.6) was purchased from LayerOne. Anhydrous DMF and tris(2,2′-bipyridyl)ruthenium(II) chloride hexahydrate were purchased from Sigma Aldrich. Reagents for porphyrin synthesis were obtained from commercial sources and were used as received. For solution preparation ultrapure water (18 MΩ cm) was used. The synthesis of TPPNH_2_ followed a literature procedure^22^ and is described in the Supporting Information along with ^1^H NMR and MALDI spectra (Figs. S1–S3). For all experiments GO or GO-CONHTPP hybrid suspensions were prepared by dispersing GO or hybrid powder in DMF or DMF-H_2_O (1:2, v/v) followed by ultrasonication for 30 min.

### Experimental apparatus

UV–Vis absorption spectra were recorded using a two-beam spectrometer Cary 100 UV–Vis scanning from 200 to 800 nm with 1 nm increments. Fluorescence spectra were recorded in the range of 500 and 800 nm on a LS 50B spectrofluorometer (Perkin Elmer) for solutions with an absorbance at the excitation wavelength lower than 0.1. Quantitative analysis of fluorescence data of graphene-containing materials is complicated by light absorption and scattering by the GO; therefore, to obtain meaningful emission data, the absorbance of free TPPNH_2_ and TPPNH_2_ attached to GO was adjusted to be identical at the excitation wavelength (0.05); in addition, the inner filter correction for the GO absorption of the excitation light was applied^23^. The fluorescence lifetimes were measured on a Fluorescence Lifetime Spectrometer (FluoTime300 from PicoQuant) with a detection system based on time-correlated single-photon counting (TCSPC). The emission decay lifetimes were measured following excitation with 408 nm diode laser. In addition, for the analysis of a fluorescence decay, an instrument response function (IRF) was obtained using Ludox solution (colloidal silica). The direct measurements of singlet oxygen emission were carried out on a FluoTime 300 fluorescence spectrophotometer with an NIR PMT detector H10330-45 (Hamamatsu) equipped with an 1000 nm long-pass filter. The samples were excited at 408 nm or 440 nm using a high repetition rate 40 MHz picosecond laser diode (LDH-405 and LDH-440, PicoQuant). Data collection was performed using a computer-mounted PCI-board multichannel scaler (NanoHarp 250, PicoQuant). The porphyrin absorbance was maintained at about 0.1 at the excitation wavelength. The time-resolved measurements (decay traces at λ = 1270 nm) were collected using a so-called “burst mode”, in which the sample was first excited using multiple pulses of the laser (1000 pulses every 25 ns giving a 25 μs excitation “burst”) to build up the population of singlet oxygen and then left to decay in the 100 μs time window of the experiment. Samples were placed in 10 mm × 10 mm quartz cells for all steady-state and time-resolved emission measurements. The quantum yields could also be determined from the recorded phosphorescence spectra; however, the time-resolved method seems to be more reliable due to the spectral overlap of different emissions at 1270 nm (e.g., for TPPNH_2_ most likely due to the 2nd order diffraction of the emission band and the characteristics of the cut-off filters used). The use of the “quasi time-gated” method discriminates the much faster emission from the relatively slow decay of the singlet oxygen phosphorescence. Details of the femtosecond transient absorption spectroscopy setup have been described elsewhere^24^. In short, the ultrafast laser system consisted of a short-pulse titanium sapphire oscillator (Mai Tai, Spectra Physics, 70 fs) followed by a high-energy titanium sapphire regenerative amplifier (Spitfire Ace, Spectra Physics, 100 fs). The 800 nm beam was split into two beams to generate: (1) a pump (*λ*_exc_ = 420 nm and 440 nm) from the optical parametric amplifier (Topas Prime with a NirVis frequency mixer) and (2) probe pulses in the UV–Vis range by using sapphire plate (Ultrafast Systems, Helios). The temporal resolution of the setup is about 300 fs. For transient UV–Vis measurements a quartz cell with 2 mm optical path of solution was used with the absorbance of about 0.2 at the excitation wavelength. The sample solution was stirred by a Teflon-coated bar. Typical pump energy was about 2 μJ. All experiments were performed at room temperature. Analysis of the transient absorption data was made using Surface Explorer software (Ultrafast Systems). The Fourier Transform Infrared Spectra (FTIR) were recorded with Bruker FT-IR IFS 66/s spectrometer (sample as tablet with KBr). NMR spectroscopy was carried out on a Bruker DPX 400 (400 MHz for ^1^H NMR) spectrometer at room temperature using an appropriate deuterated solvent. Mass spectrometry results (HRMS) were obtained using a Q-Tof Premier Waters MALDI quadrupole time-of-flight (Q-TOF) mass spectrometer equipped with a Z-spray electrospray ionization (ESI) and a matrix assisted laser desorption ionization (MALDI) sources in positive mode with *trans*-2-[3-(4-*tert*-butylphenyl)-2-methyl-2-propenylidene]malononitrile as the internal matrix. A Digital Stuart SMP10 melting point apparatus was used to determine all melting points, which are uncorrected. Samples for imaging were prepared by drop-casting solution/suspension of interest onto silicon wafer and drying in air. The morphology of samples was examined by scanning electron microscopy (SEM) Quanta 250 FEG, FEI. The measurements were performed in high vacuum using accelerating voltage 2 kV. X-ray photoelectron spectroscopy (XPS) measurements were performed using a SPECS-XPS photoelectron spectrometer. Samples for XPS measurements were prepared by dropping the dilute colloidal dispersion onto a silicon wafer and dried in the air at room temperature. Raman spectra of the samples were recorded on a LabRamHR Evolution spectrometer from Horiba Scientific coupled to a BXFM microscope (Olympus) with × 100_Vis_LWD objective, using a Nd:YAG laser with a wavelength of 532 nm. Samples for Raman measurements were prepared by drop-casting a solution of TPPNH_2_ and a suspension of GO and GO-CONHTPP onto silicon wafers and drying in air.

### Computational details

We followed closely the protocol validated in our previous studies^25–27^. Briefly, the GO was represented as C_59_O_26_H_26_ Lerf-Klinowski-type model that was shown to be robust in analyzing non-covalent functionalization with porphyrins^27^. The structures of free TPPNH_2_ and two hybrids (covalent GO-CONHTPP and noncovalently interacting GO/TPPNH_2_) were optimized at the density functional theory (DFT) level using BP86 functional^28^ augmented with D3BJ^29,30^ and gCP^31^ corrections to improve the description of dispersion interactions and to compensate for the basis set superposition error, respectively. This setup is abbreviated as BP86 + D3gCP. The structures obtained were subjected to numerical second derivative calculations to confirm their character as true energy minima at the potential energy surface. Single-point calculations at optimized structures were performed using BHLYP functional^32^ to decrease electron delocalization error and provide data directly comparable to our previous studies. The basis set applied was def2-TZVP^33^. All DFT calculations were performed using ORCA 4.2.0 program^34^. The numerical overlap between selected orbitals was calculated with Multiwfn 3.6^35^.

## Results and discussion

### Synthesis and characterization

**Figure 1:**
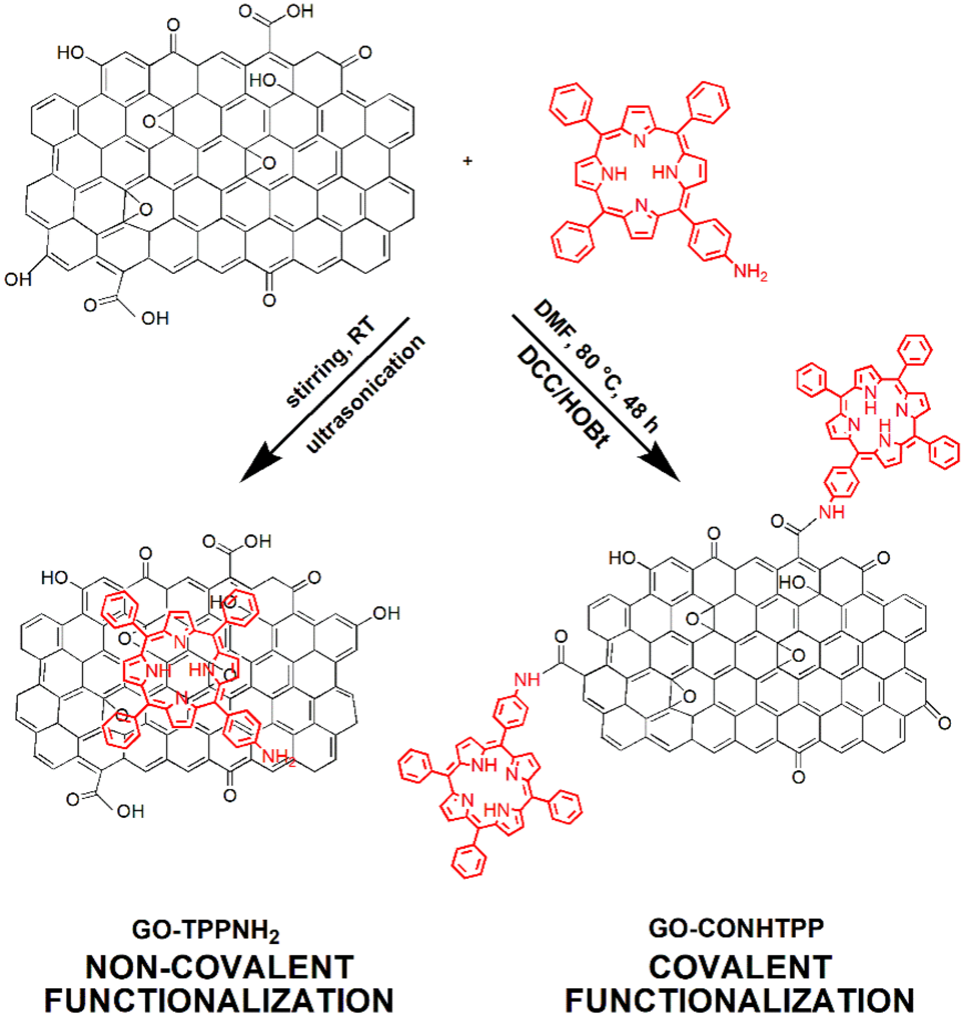
Schematic illustration for the preparation of GO with covalently and non-covalently linked TPPNH_2_; DCC—*N*,*N*′-dicyclohexylcarbodiimide, HOBt—1-hydroxybenzotriazole.

The covalent linkage of GO and TPPNH_2_ was obtained by direct coupling of the GO carboxylic groups with the amino group of TPPNH_2_ to form an amide in one-step^36–40^, as illustrated in Fig. 1. For comparison, GO non-covalently functionalized with TPPNH_2_ was prepared by simple mixing and sonication of GO and TPPNH_2_ dispersions.

**Figure 2:**
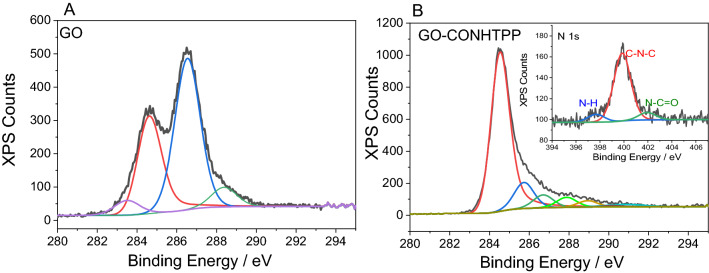
XPS spectra of C1s for (**A**) GO and (**B**) GO-CONHTPP and N1s for GO-CONHTPP in the inset.

GO-CONHTPP nanohybrid was characterized via FTIR, Raman and XPS spectroscopy and scanning electron microscopy (SEM) (Fig. 2 and Figs. S4–S6) which provided evidences for successful functionalization of GO with TPPNH_2_. In the FTIR spectrum of the covalent nanohybrid signals characteristic for porphyrin are noticed (red stars in Fig. S4), among others at 800 cm^−1^ and 734 cm^−1^, which were not present in the spectrum of GO itself. At the same time, bands characteristic for the N–H stretching vibrations in the NH_2_ amino groups are absent. Additionally, the signal at 1734 cm^−1^ characteristic of the carbonyl in carboxylic groups disappears, and a new band at 1650 cm^−1^ appears, which can be attributed to amide C=O stretching. Likewise, a new band at 1450 cm^−1^, not observed for free TPPNH_2_ and GO, can be attributed to the stretching vibrations of the C–N bonds in amide groups. These results indicate that the TPPNH_2_ molecules are covalently bound to the graphene oxide via amide linkages.

In the Raman spectra for the covalent nanocomposite, Raman peaks characteristic of the porphyrin macrocycle are visible in the spectra (Fig. S5). No shifts of the porphyrin signals after TPPNH_2_ attachment to the GO sheets were observed, which indicates lack of the close contact (π–π stacking) between the surface of the GO sheets and the porphyrin macrocycles, in accordance with further presented data.

X-ray photoelectron spectroscopy (XPS, Fig. 2) shed more light on the structure of starting material GO and the functionalized material GO-CONHTPP. The oxygen content in GO is ca. 30.7 atom %, which suggests a high degree of oxidation of the starting material. The XPS spectra of C1s for GO were deconvoluted into four peaks, 283.5, 284.6, 286.5, 288.4 eV, corresponding to the characteristic peaks of C–C, C=C, C–O, O–C=O, respectively (Fig. 2A). Compared with GO, the oxygen-containing functional group (O–C=O, C=O, C–O–C, and C–OH) peaks of GO-CONHTPP decrease sharply, while the sp^2^ C=C bond carbon peak (284.5 eV) increases significantly. The total C/O atomic ratio for GO-CONHTPP increased to 7.7 from 2.1 of GO, clearly demonstrating a reduction of GO during the functionalization process.

The binding of TPPNH_2_ molecules to GO was further confirmed by the increased content of nitrogen by 0.3% in comparison to the GO material subjected to the same synthetic procedure in the absence of porphyrin. This relates to a calculated porphyrin content in the hybrid material of 2.7% by weight. Due to the limited number of carboxylic groups on GO, the functionalization degree is low and comparable to other porphyrins attached to GO via an amide bond^41^.

A typical N1s XPS for free base porphyrins shows two components arising from the macrocycle’s two inequivalent nitrogen atoms, which are usually separated by approximately 2 eV^42,43^. The N1s spectra were deconvoluted into three peaks (inset in Fig. 2B). The peak at 397.7 eV originates from the iminic nitrogen, while the one at 399.9 eV is attributed to the pyrrolic nitrogen. The third peak at 401.9 eV is assigned to N–C=O. The presence of the latter peak indicated that TPPNH_2_ was linked to the GO via amide bonds.

### Photophysical characterization

#### Absorption properties

**Figure 3:**
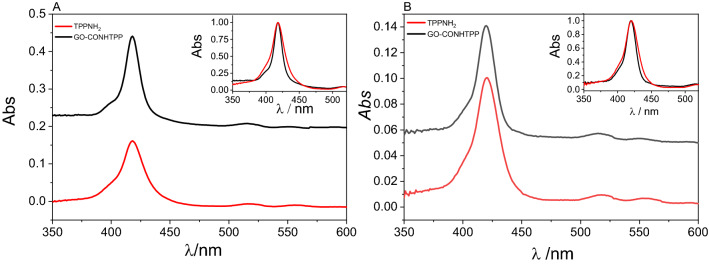
Absorption spectra of free TPPNH_2_ (red curve), TPPNH_2_ covalently bound to GO (black curve) in: (**A**) DMF, (**B**) DMF-H_2_O (1:2 v/v). Insets: Normalized absorption spectra of TPPNH_2_ and GO-CONHTPP.

The absorption spectra of TPPNH_2_ in DMF and DMF-H_2_O (1:2, v/v) exhibited the expected porphyrin-specific bands, i.e. the Soret and four Q-bands (Fig. S7). In DMF-H_2_O (1:2, v/v) the Soret band of TPPNH_2_ is red-shifted by 2 nm compared to DMF, and the band is slightly broadened. The presence of the characteristic Soret and Q bands in UV–Vis spectrum of GO-CONHTPP validates the existence of porphyrin in the nanocomposites (Fig. 3).

Interestingly, no shift of the TPPNH_2_ Soret band at 419 nm is observed upon covalent binding to the GO sheets, which is in agreement with the report by Xu et al.^44^. The only difference in the UV–Vis spectra of TPPNH_2_ attached to the GO versus free TPPNH_2_ is a slight broadening of the Soret band of the latter. The broadening of the TPPNH_2_ Soret band may indicate aggregation of the porphyrin molecules, which is presumably inhibited once TPPNH_2_ is bound to GO.

The absorption spectra of GO-CONHTPP in DMF-H_2_O (1:2 v/v) also largely resemble the UV–Vis spectra of the free porphyrin (Fig. 3B). The lack of noticeable changes in TPPNH_2_ absorption upon covalent functionalization to GO might be explained by the rigid amide bond preventing TPPNH_2_ molecules from interacting via π–π stacking with the GO surface they are linked to. Other related covalent systems, which were formed via amide or ester linkage between –COOH at the edges of GO surface and porphyrin, also exhibited lack of significant change in the Soret band position^41,45^. However, the position of the Soret band was found to be red-shifted for covalent hybrids when porphyrin was used with a modified longer amide axial ligand or when porphyrin was attached directly to the GO surface using an epoxy group present on the surface^46,47^. These examples indicate that π–π stacking between hybrid components is essential to observe changes in the UV–vis spectra of the porphyrin. In our study the lack of π–π stacking between TPPNH_2_ molecules and the GO surface has been further confirmed by calculating theoretically the structure of the hybrid material (vide infra).

**Figure 4:**
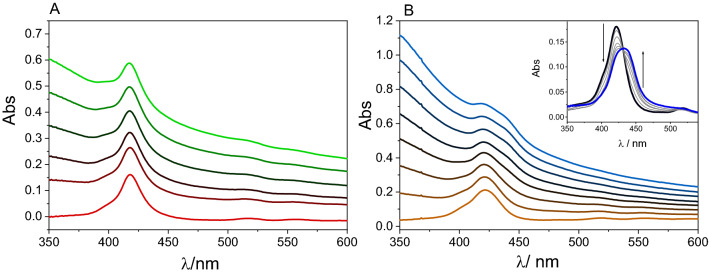
Absorption spectra recorded for 3 mL of 0.7 µM solution of TPPNH_2_ in: (**A**) DMF with GO dispersion (0–0.066 mg mL^−1^), (**B**) DMF-H_2_O (1:2, v/v) with GO dispersion (0–0.1 mg mL^−1^). The inset highlights the Soret band region with the spectra corrected for GO absorption.

To compare the interactions between TPPNH_2_ bound covalently or non-covalently to the GO, the optical absorption spectra of a series of TPPNH_2_ solutions in which GO was gradually added to a TPPNH_2_ solution were measured (Fig. 4). No significant spectroscopic changes occur when titrating the TPPNH_2_ solution with GO suspension in DMF (Fig. 4A). After centrifuging the mixture of TPPNH_2_ and GO in DMF, virtually all TPPNH_2_ was still present in the supernatant, and GO was collected as the precipitate. This indicates that the interaction between TPPNH_2_ and GO in DMF is weak.

Conversely, changes in the UV–Vis spectra were noted when titrating in DMF-H_2_O (1:2, v/v) (Fig. 4B). The intensity of the original Soret band at 421 nm gradually decreased, and a new Soret band appeared at 440 nm, together with the presence of isosbestic point at 430 nm (inset in Fig. 4B). The Soret band was found to be red-shifted by 19 nm for TPPNH_2_ adsorbed on the GO surface and slightly broadened in comparison to free TPPNH_2_ (Fig. S8).

The bathochromic shift observed upon nanohybrid formation could be attributed to a decrease in the meso*-*phenyl substituents tilt angle adsorbed on the GO sheet as described previously for related hybrids porphyrin-GO^25–27^. Interestingly, three of the four Q bands disappeared for TPPNH_2_ adsorbed on the GO surface in DMF-H_2_O (1:2, v/v), and a new broad band was observed at 659 nm (Fig. S8). The presence of an intense long-wavelength band can be explained by partial charge transfer from TPPNH_2_ to the GO sheet what is supported by our theoretical calculations (vide infra) and similarity with literature data on porphyrin radical cation spectra^12,48,49^.

Centrifugation of the mixture of TPPNH_2_ and GO allows efficient nanohybrid isolation (Fig. S9). Based on the minor peak attributed to porphyrin in the UV–Vis spectrum of the supernatant, it is clear that all of the nanohybrids obtained were successfully collected as a precipitate. This experiment confirms that GO can be successfully functionalized non-covalently with TPPHN_2_ in DMF-H_2_O (1:2, v/v) by simply mixing solutions of the two components. Based on this experiment, it was estimated that the maximum amount of TPPNH_2_ that can be adsorbed on the GO surface is 1.3%. This is similar to values reported for other non-covalent hybrids of GO with neutral porphyrins, such as 5,10,15,20-tetra(hydroxyphenyl)porphyrin or its zinc derivative^13,25,26^.

In summary, the presence of water favors the formation of stable non-covalent GO-TPPNH_2_ hybrids. While the absorption spectrum of porphyrin remains unchanged for GO-CONHTPP compared to free porphyrin, for TPPNH_2_ adsorbed non-covalently to GO, the absorption spectra differ significantly in DMF-H_2_O (1:2, v/v). Our results demonstrate that the type of GO functionalization—covalent vs. non-covalent—affects the ground state electronic structure of the porphyrin.

The structure of covalent and non-covalent hybrid materials predicted by theoretical calculations revealed that close proximity and π–π stacking interaction of the porphyrin molecule with the GO sheet is only possible for the non-covalent functionalization. Based on the results described above, it can therefore be concluded that π–π stacking interaction is required to modify the electronic structure of the porphyrin which is manifested by the changes in the electronic absorption spectra. This is consistent with previous reports demonstrating that red-shift of the Soret band for covalent hybrids is observed only when porphyrin with a modified longer amide axial ligand was used or when the porphyrin was attached directly to the GO surface using an epoxy group present on the surface^46,47^.

**Figure 5:**
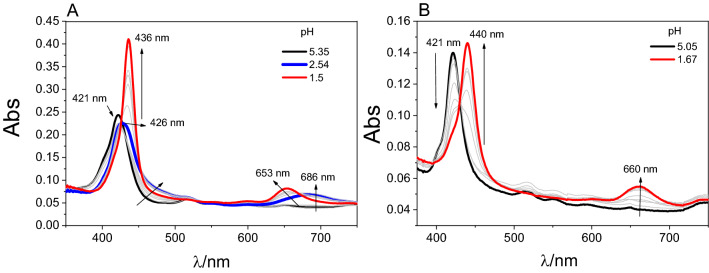
Absorption spectra recorded during the addition of 1 M HCl to a solution of: (**A**) TPPNH_2_ and (**B**) GO-CONHTPP in DMF-H_2_O (1:2, v/v).

Spectroscopic measurements as a function of pH were used to provide additional evidence for the covalent attachment of TPPNH_2_ to GO. Changes in TPPNH_2_ towards acidic pH introduce positive charge to neutral TPPNH_2_ in two steps corresponding to protonation of peripheral amine -NH_2_ and the imine nitrogens in the core (Fig. S10). Spectra of acid-titration of TPPNH_2_ clearly demonstrates two well-separated stages (pK_a_ 3.01 and 2.01) both indicated by isosbestic points (Fig. 5 and Fig. S11).

Titration of hybrid GO-CONHTPP with acid gave only a single isosbestic point with the pK_a_ of 2.36; hence, only core protonation occurred (Fig. 5B and Fig. S12). This experiment proves that there is no free amine group in TPPNH_2_ when it is attached to the GO and no free porphyrin remains in the hybrid after synthesis.

#### Emission properties

**Figure 6:**
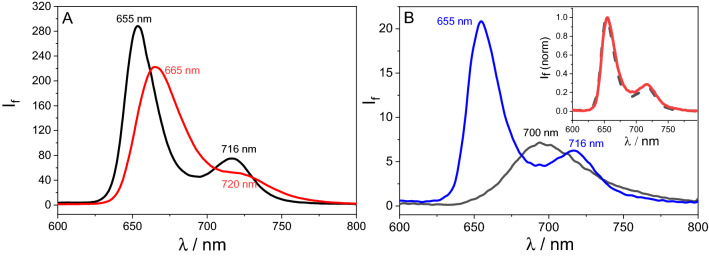
(**A**) Emission properties of TPPNH_2_ (red) and GO-CONHTPP (black) in DMF, λ_exc_ = 419 nm. (**B**) Fluorescence spectra registered for TPPNH_2_ after the addition of 20 µL of 1 M HCl at two excitation wavelengths: 419 nm (blue) and 450 nm (black). Inset: Normalized fluorescence spectra of GO-CONHTPP in DMF and TPPNH_2_ at excitation wavelength 419 nm in acidified DMF.

To investigate the excited state interactions of TPPNH_2_ and graphene oxide in the hybrid, the fluorescence spectra of the TPPNH_2_ and GO-CONHTPP hybrid were compared. Surprisingly, compared to TPPNH_2_, GO-CONHTPP demonstrates even higher fluorescence intensity after applying the appropriate inner filter correction (the intensity was lower for uncorrected spectra). This is contrary to previously reported data for TPPNH_2_ covalently functionalized with graphene^41,44^. This difference may arise either from difficulties in the quantitative analysis of emission data for graphene-based materials or from different electronic interactions between the components of the hybrid materials. The interactions between TPPNH_2_ and GO depend on the exact structure of GO, which strongly depends on the synthesis protocol^41,44^. In both studied solvents for GO-CONHTPP, the emission at 665 nm is blue-shifted by ca. 10 nm compared to free TPPNH_2_, consistent with previous findings (Fig. 6A)^41,44^. Porphyrins are known to aggregate strongly^50–54^, and therefore, the decrease in fluorescence intensity in comparison with GO-CONHTPP and the shift in the emission of free TPPNH_2_ can be explained by aggregation. As presented in Fig. S13, the fluorescence excitation spectrum recorded for GO-CONHTPP and TPPNH_2_ solution corresponds to their absorption spectrum, although the latter is slightly broadened. To verify the hypothesis of free TPPNH_2_ aggregation, we measured the emission spectra of free TPPNH_2_ in DMF after addition of 20 µL of 1 M HCl which results in an equilibrium state between neutral and protonated porphyrin (Fig. 6B). Excitation at 450 nm leads to recording the emission spectra of protonated TPPNH_2_, which is broad with the maximum at 700 nm^55^. Upon excitation at 419 nm, emission from neutral TPPNH_2_ present in acidic DMS was observed with maximum at 655 nm which matches the emission spectrum of the nanocomposites GO-CONHTPP (see inset in Fig. 6B). The addition of a small amount of HCl to TPPNH_2_ solution in DMF destroys the aggregates due to the repulsive interaction of the protonated molecules. The molecules become non-planar and hence cannot aggregate^56^. Therefore, the emission spectra of neutral TPPNH_2_ molecules in acidic DMF coincide with those recorded for the covalent GO-CONHTPP composites in both DMF and DMF-H_2_O (1:2, v/v).

**Figure 7:**
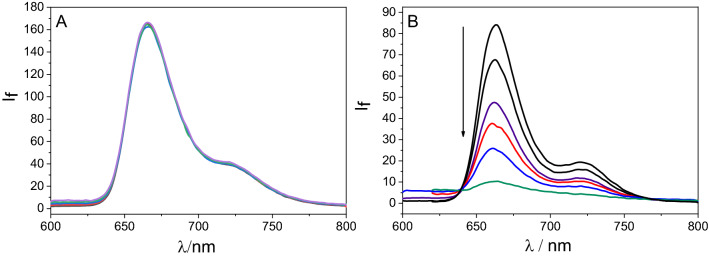
The emission spectra of TPPNH_2_ recorded during addition of a suspension of GO (0–0.067 mg mL^−1^). In: (**A**) DMF, λ_exc_ = 419 nm, (**B**) DMF-H_2_O (1:2, v/v), λ_exc_ = 430 nm. Spectra were corrected for the inner filter effects I and II.

For comparison, the interaction of the singlet excited state of TPPNH_2_ non-covalently attached to GO sheets was investigated by emission spectroscopy. Upon correcting the experimental data for absorption of the excitation light by GO and the absorption of TPPNH_2_ emission by GO, it was found that in DMF no fluorescence quenching occurred at all (Fig. 7A). In contrast a significant decrease in the TPPNH_2_ fluorescence intensity was observed with increasing GO concentration in DMF-H_2_O (1:2, v/v) but with no observable changes in the peak shape and position (Fig. 7B). Moreover the fluorescence excitation spectrum of TPPNH_2_ after the addition of GO, corresponded to the absorption spectrum of unbound TPPNH_2_ (Fig. S15). This indicates that the non-covalent nanohybrid TPPNH_2_-GO is not emissive or only a very weakly emissive material in DMF-water mixture. A possible reason for the non-emissivity of TPPNH_2_-GO complex is the existence of a competitive very fast non-radiative process that deactivates singlet excited state.

In summary, firstly, steady-state absorption and emission measurements show that the interaction strength between TPPNH_2_ and GO can be modulated by the choice of solvent system. Secondly, data from fluorescence spectra indicate that there is no interaction of the singlet excited state of the porphyrin with GO in covalent composites in both solvent systems studied, similarly like for the in non-covalent composites in DMF.

**Figure 8:**
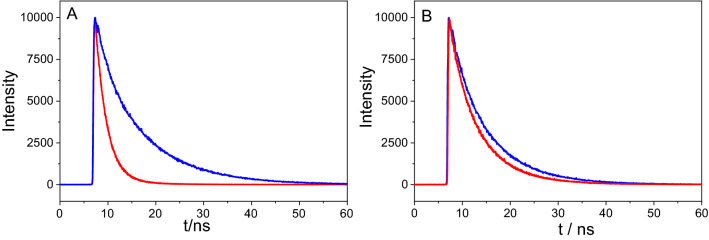
Decay of a fluorescence recorded for TPPNH_2_ (red) and GO-CONHTPP (blue) in: (**A**) DMF and (**B**) DMF-H_2_O (1:2, v/v) (*λ*_ex_ = 405 nm, *λ*_em_ = 650 nm).

To further probe the possible interaction of the singlet excited state of porphyrin functionalized covalently and non-covalently with GO we used time-correlated single photon counting. The fluorescence lifetime of TPPNH_2_ in DMF extracted from the fluorescence decay was approximately 2.4 ns and 8.6 ns for free TPPNH_2_ and GO-CONHTPP, respectively (Fig. 8A). The corresponding values in DMF-H_2_O (1:2, v/v) were 5.2 ns and 8.1 ns for free TPPNH_2_ and GO-CONHTPP, respectively (Fig. 8B). The longer fluorescence lifetime for GO-CONHTPP is in agreement with the higher emission intensity measured in steady-state experiment. A possible reason is that unbound TPPNH_2_ molecules in solution are prone to form aggregates, whereas covalent attachment to GO prevents formation of aggregates, at least at low concentration. It is well known that porphyrin aggregates are characterized by lower emission intensity or even lack of fluorescence in comparison to monomers^57^. To clarify this point further, we performed time-resolved emission measurements with different TPPNH_2_ concentrations which revealed a strong dependence of the TPPNH_2_ fluorescence lifetime on its concentration (Fig. S16). Therefore, the formation of TPPNH_2_ aggregates hinders direct comparison of the emission properties of the free monomer of TPPNH_2_ and covalently attached to GO.

The possible interaction of the TPPNH_2_ singlet excited state with GO was further examined by monitoring the emission decay profiles of TPPNH_2_ in the presence of different GO concentrations. When GO was added to TPPNH_2_, the lifetime remained practically unaltered and insensitive to its concentration (Fig. S17). Lack of observable change in the singlet excited state lifetime of free TPPNH_2_ excludes dynamic quenching by GO. Taking into account results from both steady-state and time-resolved emission experiments, it can be stated that the observed emission quenching of TPPNH_2_ upon addition of GO is due to static quenching.

### Femtosecond transient absorption spectroscopy

**Figure 9:**
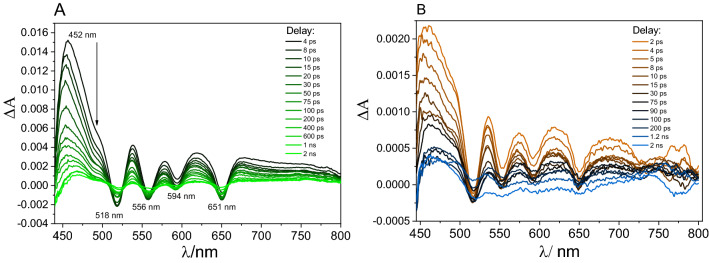
Transient absorption spectra registered at various time delays for: (**A**) TPPNH_2_ and (**B**) GO-CONHTPP in DMF-H_2_O (1:2, v/v) following the 422 nm laser excitation.

Of particular interest was the investigation of a possible electron transfer process from the singlet excited state of the porphyrin to graphene oxide sheets, as this process is crucial for potential applications in solar energy conversion. The singlet excited state differential absorption measured immediately after the 422 nm laser pulse excitation of unbound TPPNH_2_ showed strong absorption around 450–500 nm and a Q-band bleaching, consistent with the Q band position observed in the UV–Vis absorption spectra (Fig. 9A).

Kinetic profile analysis revealed the presence of two exponential decays of the transient absorption signal at 452 nm with time constants of 14 and 206 ps (Fig. S18). The residual absorption observed as a small offset which does not vanish over the entire experimental time window (3 ns) (on Fig. S18 depicted for 452 nm and 520 nm) can be attributed to the triplet excited state. The decay profiles from fs transient absorption spectroscopy do not match the data obtained by the TCSPC technique. However, the femtosecond experiments were performed at 10 times higher porphyrin concentrations than in the TCSPC experiment. As discussed above, the fluorescence decay obtained by TCSPC method strongly varies with the porphyrin concentration. Therefore, the observed fast decays on the fs transient absorption spectroscopy could be attributed to the aggregates present in the sample.

The transient absorption and kinetic profiles of GO-CONHTPP covalent composites showed spectral features very similar to those registered for the free dye (see Fig. 9B) what indicates that electron transfer in the covalent hybrid material is very unlikely.

**Figure 10:**
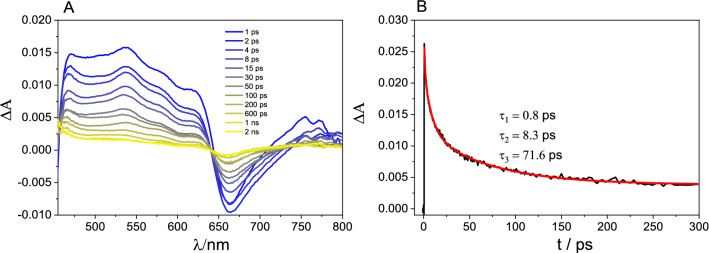
(**A**) Transient absorption spectra registered at various time delays and (**B**) absorption time profile probed at 550 nm measured for TPPNH_2_ in the presence of GO (GO concentration 0.4 mg mL^−1^) in DMF-H_2_O (1:2, v/v) following the 440 nm laser excitation. Transient absorption spectra and kinetics are corrected for GO contribution.

An analogous transient absorption (TA) experiment was performed when GO was added to a TPPNH_2_ solution in DMF-H_2_O (1:2, v/v) (Fig. 10A). The spectra presented in Fig. 10A were corrected for the transient absorbance of GO (Fig. S19)^58^. Clearly, the TA spectra of TPPNH_2_ non-covalently functionalized with GO (Fig. 10A) recorded just after excitation do not resemble the TA spectra obtained for free TPPNH_2_ (Fig. 9A).

As presented in Fig. 10A, the formation of a broad band in the region of 450–650 nm was detected. This broad positive band can be attributed to the porphyrin radical cation based on the similarity to reported spectra for the related systems^49,59^. The presence of the TPPNH_2_ radical cation just after the laser pulse in the system, where TPPNH_2_ was simply mixed with GO, provides evidence for fast photoinduced electron transfer from the porphyrin to GO. The absorption profile at 550 nm attributed to the TPPNH_2_ radical cation disappears with time constants of 0.8 ps, 8.3 ps, and 71.6 ps, which can be explained by the fast back electron transfer (Fig. 10B). This is supported by the bleach recovery at 660 nm, which follows the same kinetics as the transient absorption decay at 550 nm. The three exponential decays of the TPPNH_2_ radical cation signal presumably reflect various TPPNH_2_ aggregates in GO nanoassemblies that may influence the rate constant of back electron transfer. Note the residual signal of about 15% in all kinetic profiles. This offset might be explained by the presence of TPPNH_2_ radical cations, which did not undergo back electron transfer during the probed time window of 3 ns. The presence of long-lived charge-separated states indicates possible solar energy conversion systems applications.

### Singlet oxygen generation

**Figure 11:**
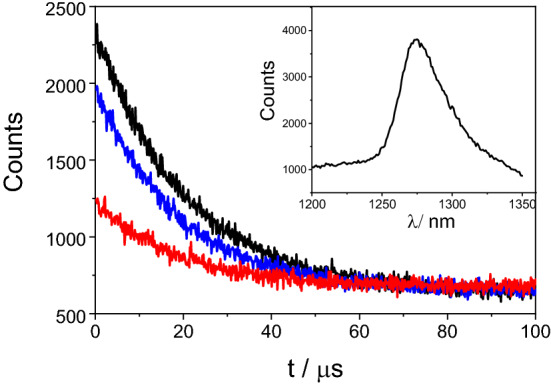
Decay curves of the singlet oxygen generated by free TPPNH_2_ (black), GO-CONHTPP (blue) and a non-covalent mixture of TPPNH_2_ and GO (red) in DMF (λ_exc_ = 408 nm, λ_mon_ = 1270 nm, collection time: 10 min). Inset: phosphorescence spectrum of ^1^O_2_ measured for TPPNH_2_ in DMF.

Evaluation of the ability of porphyrin/GO nanohybrids to produce reactive oxygen species can be valuable for predicting in vitro activity against cancer cells or microorganisms^60^. Optically diluted solutions of free TPPNH_2_, GO-CONHTPP, and the mixture of TPPNH_2_ and GO in DMF were excited at 408 nm, and the photogeneration of singlet oxygen was measured by detecting its phosphorescence decay at λ = 1270 nm^61^. The singlet oxygen quantum yields (ΦΔ) in DMF were determined by comparison of the amplitudes of the decay curves collected for 10 min with that recorded for tris(2,2′-bipyridine)ruthenium(II) (ΦΔ = 0.69, DMF), used as a standard (Fig. 11)^62^. Tail fitting yielded a lifetime of 26 µs, which agrees with published literature values for the lifetime of single oxygen in DMF^63^.

The GO-CONHTPP was found to sensitize the production of singlet oxygen with a quantum efficiency (ΦΔ = 0.20) comparable to that of free TPPNH_2_ (ΦΔ = 0.26). This supports the absence of GO interaction with the singlet excited state of porphyrin, which could suppress the efficient intersystem crossing and triplet state formation. In DMF-H_2_O (1:2, v/v), the singlet oxygen decay amplitude was identical for free TPPNH_2_ and GO-CONHTPP (Fig. S20). Noteworthy, TPPNH_2_ attached non-covalently to GO in DMF is a photosensitizer with significantly lower singlet oxygen quantum yield (ΦΔ = 0.08) relative to free TPPNH_2_. For the same non-covalent TPPNH_2_/GO composite in DMF-H_2_O (1:2, v/v) no singlet oxygen production was detected, confirming the involvement of electron transfer interactions between excited porphyrin and GO. Maintaining the singlet oxygen quantum efficiency by TPPNH_2_ upon covalent functionalization to GO is an important outcome useful for designing graphene-based photosensitizers for photodynamic/photothermal therapy.

**Figure 12:**
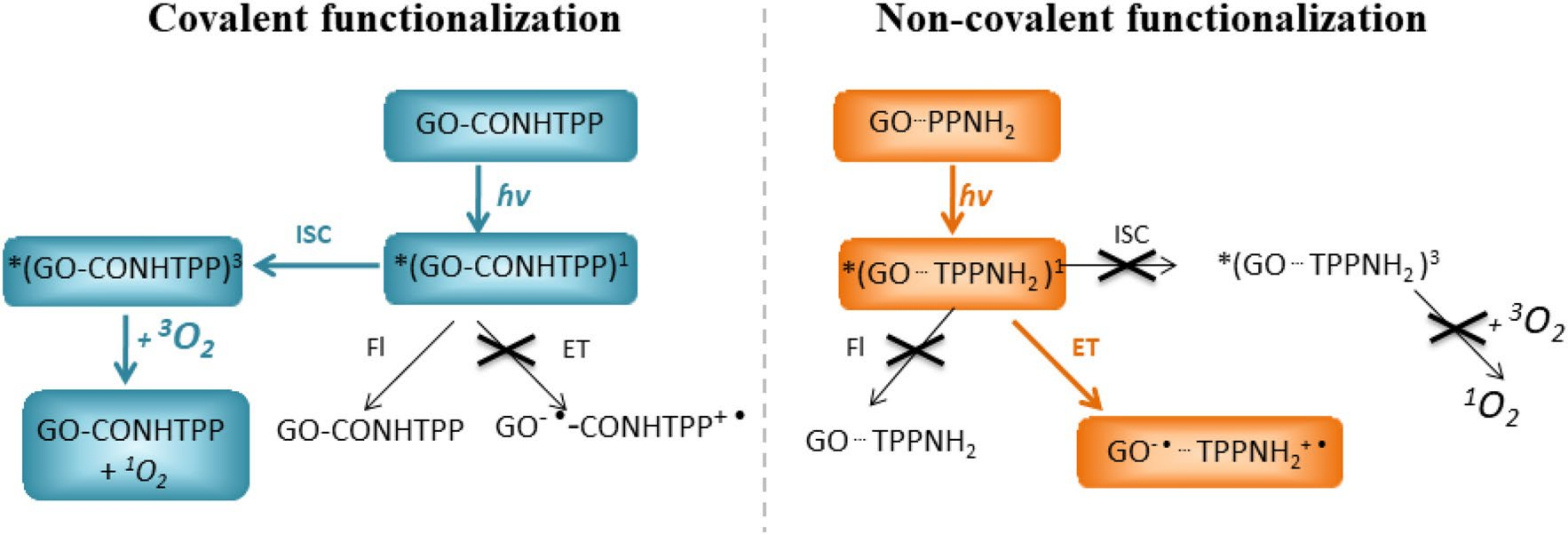
Possible deactivation paths of the excited states of GO-CONHTPP and GO-TPPNH_2_ discussed in text (*Fl* fluorescence, *ET* electron transfer). Deactivation paths important for practical application were marked in blue and orange for GO-CONHTPP and GO-TPPNH_2_, respectively.

In summary, our spectroscopic experiments revealed significant differences between the deactivation pathways of the excited state of porphyrins depending on the type of functionalization with GO (Fig. 12). For GO-TPPNH_2_ fast electron transfer from the singlet excited state of porphyrin to GO prevents the formation of the triplet excited state. As a result the ^1^O_2_ generation was suppressed. In contrary, for GO-CONHTPP the ^1^O_2_ generation yields was comparable to free TPPNH_2_ what reflects weak interaction of the GO with the excited state of the porphyrins.

### Quantum chemical calculations

**Figure 13:**
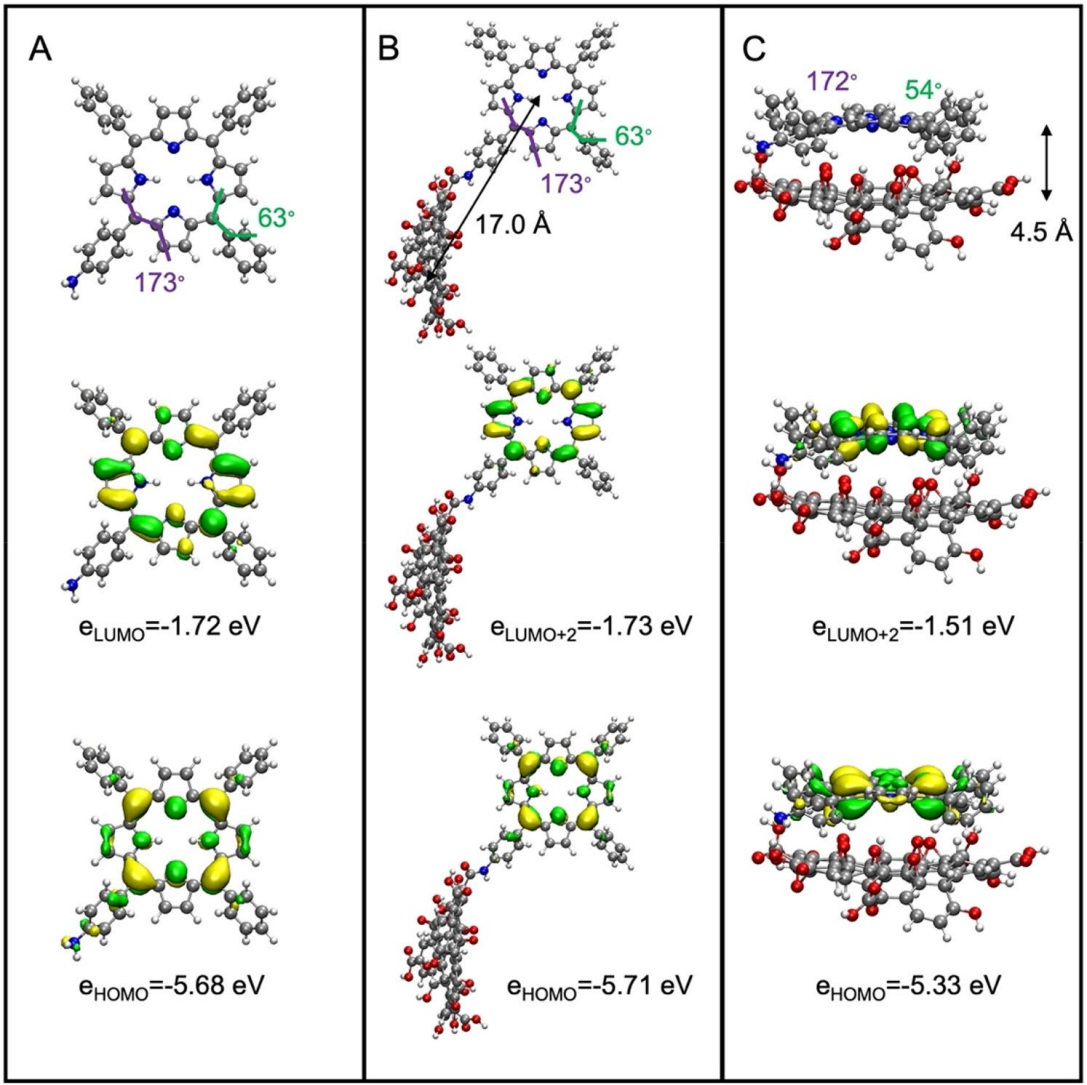
Calculated structures (BP86 + D3gCP/def2-TZVP; top) and selected frontier molecular orbitals strongly localized in the porphyrin moiety along with corresponding orbital energies (middle and bottom) for: (**A**) TPPNH_2_, (**B**) GO-CONHTPP, and (**C**) GO/TPPNH_2_ noncovalent hybrid. Molecular orbitals were computed at BHLYP/def2-TZVP level and drawn as ± 0.03 a.u. isosurfaces. Key dihedral angles are marked in color (purple and green), the distances between centers of mass between GO and porphyrin moieties are denoted by black arrows.

We have recently validated a molecular model for graphene oxide that has been successfully used to gain insights into electronic structure changes upon non-covalent nanohybrids formation with various porphyrins^25–27^. We note that carboxylic groups are placed at the GO system’s terminal (equatorial) positions and can participate in the amide bond formation with a porphyrin. Such properties allow us to directly compare the electronic structure of the covalently and non-covalently bound hybrids studied in the present work. The free porphyrin has an expected structure and HOMO–LUMO gap (3.96 eV) similar to previously studied systems (Fig. 13)^25–27^. The GO-CONHTPP hybrid features an amide bond in an expected *trans* configuration. Porphyrin and GO moieties are spatially well separated (distance between centers of mass of 17.0 Å) and oriented perpendicular to each other. The inspected dihedral angles of the porphyrin moiety are virtually indistinguishable from free TPPNH_2_; this is reflected in almost unchanged energies of the corresponding molecular orbitals.

In contrast, the non-covalent hybrid displays stacking that favors the cofacial orientation of hybrid components. Consequently, the subsystems are close to each other (distance between centers of mass of 4.5 Å), and the TPPNH_2_ molecule flattens as the C–C–C–C dihedral angles (green in Fig. 13), describing the out-of-plane rotation of the phenyl substituents decreases compared to free TPPNH_2_. Such geometrical changes significantly impact the energies of the frontier molecular orbitals of the TPPNH_2_ molecule. We observed a decrease in the energy difference between the corresponding orbital pairs compared to isolated porphyrin (e.g., from 3.96 to 3.82 eV), which explains the experimentally observed red-shift of the Soret band.

Consistent with our previous studies, the HOMO is strongly localized on porphyrin, while the first unoccupied MO is GO-centered. Thus, one might expect a low-energy charge-transfer excited state for porphyrin. However, we expect the probability of charge transfer to be very different between the two studied hybrids. We, therefore, computed an overlap integral *S*_H/L_ between HOMO/LUMO pairs for covalently bound and non-covalently interacting hybrids to quantify this issue. This was negligibly small for the former (*S*_H/L_ = 0.002), while it was an order of magnitude more significant for the latter (*S*_H/L_ = 0.069). Thus, the probability of electron transfer, which is proportional to *S*_H/L_, decreases when the porphyrin is covalently anchored to the GO.

## Conclusions

**Figure 14:**
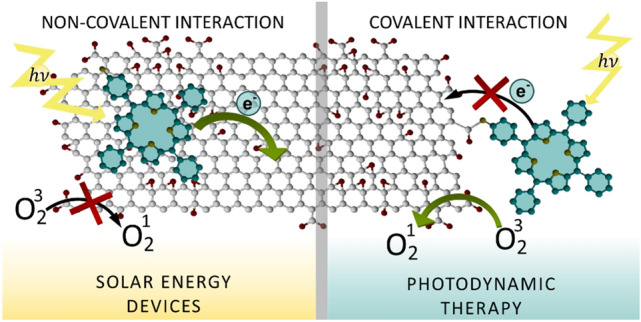
Schematic representation of the influence of the porphyrin/GO hybrid functionalization type on their interaction with light.

Herein, it was demonstrated that while TPPNH_2_ can be successfully assembled on the GO surface via either covalent or non-covalent bonding, the properties of these two types of hybrid materials differ significantly. The covalent attachment of TPPNH_2_ was achieved through amide formation at the periphery of GO sheets and the hybrid material was fully characterized by FTIR, XPS, Raman spectroscopy, and SEM. Spectroscopic measurements together with theoretical calculations demonstrated that assembling TPPNH_2_ on the GO surface in DMF-H_2_O (1:2, v/v) via non-covalent interactions causes changes in the absorption spectra of porphyrin, as well as efficient quenching of its emission. Interestingly, covalent binding to GO does not affect notably neither the porphyrin absorption nor its fluorescence. Our results clearly demonstrate that the type of functionalization influences the perturbation of the ground state electronic structure of TPPNH_2_ by GO. The structure of the covalent and non-covalent hybrid materials predicted by theoretical calculations indicates that close proximity and π–π-stacking of the porphyrin molecule with the GO sheet is possible only for the non-covalent functionalization. Therefore, it can be concluded that π–π-stacking interaction is required to modify the ground state electronic structure of porphyrin in the assembly. Secondly, femtosecond pump–probe experiments revealed that only the non-covalent assembly of TPPNH_2_ and GO enhances the efficiency of the photoinduced electron transfer from porphyrin to GO. Our results clearly demonstrate that although covalent functionalization prevents aggregation of the porphyrin molecules the electronic interaction of porphyrin and GO both in the ground and excited state, is limited. The negligible excited state interaction of porphyrin with GO in the covalent hybrid was reflected in its ability to generate singlet oxygen comparable to free TPPNH_2_, thus indicating the feasibility of covalent hybrid materials for photodynamic/photothermal therapy (Fig. 14).

In summary, our results demonstrate that by covalently attaching TPPNH_2_ to the GO, a material with sufficient yield of singlet oxygen generation is obtained, whereas non-covalent assembly leads to a material exhibiting efficient PET properties. This study provides valuable knowledge for developing highly efficient nanomaterials for desired applications.

## Supplementary Information

Supplementary Information.

## Data Availability

All data generated or analyzed during this study are included in this published article (and its Supplementary Information files).
