# Supplementary material for: Understanding structure–properties relationships of porphyrin linked to graphene oxide through π–π-stacking or covalent amide bonds
Source: Sci Rep. 2022 Aug 4;12:13420. doi: 10.1038/s41598-022-16931-8 (PMC9352710; doi:10.1038/s41598-022-16931-8)
Supplement: Supplementary file 1 — Supplementary Information. [file 41598_2022_16931_MOESM1_ESM.docx]

**Supporting Information**

**Understanding structure–properties relationships of porphyrin linked to graphene oxide through π-π-stacking or covalent amide bonds**

Anna Lewandowska-Andralojc^a,b,*^, Ewelina Gacka^a,b^, Tomasz Pedzinski^a,b^, Gotard Burdzinski^c^, Aleksandra Lindner^d^, Jessica M. O’Brien^e^, Mathias O. Senge^e,f^, Alexandra Siklitskaya^g^, Adam Kubas^g^, Bronislaw Marciniak^a,b^, Justyna Walkowiak-Kulikowska^a^

*^a^Faculty of Chemistry, Adam Mickiewicz University, Uniwersytetu Poznanskiego 8, 61-614 Poznan, Poland*

*^b^Center for Advanced Technology, Adam Mickiewicz University, Uniwersytetu Poznanskiego 10, 61-614 Poznan, Poland*

*^c3^Faculty of Physics, Adam Mickiewicz University, Uniwersytetu Poznanskiego 2, 61-614 Poznan, Poland*

*^d^Helmholtz-Zentrum Dresden-Rossendorf, Institute of Ion Beam Physics and Materials Research, Bautzner Landstraße 400, 01328 Dresden, Germany*

*^e^School of Chemistry, Chair of Organic Chemistry, Trinity Biomedical Sciences Institute, Trinity College Dublin, The University of Dublin, 152-160 Pearse Street, Dublin 2, Ireland*

*^f^Institute for Advanced Study (TUM-IAS), Focus Group – Molecular and Interfacial Engineering of Organic Nanosystems, Technical University of Munich, D-85748 Garching, Germany*

*^g^Institute of Physical Chemistry, Polish Academy of Sciences, Kasprzaka 44/52, 01-224 Warsaw, Poland*

# Synthesis of 5-(4-aminophenyl)-10,15,20-triphenylporphyrin:

TPP (300 mg, 0.489 mmol) was dissolved in TFA (30 mL). Sodium nitrite (60 mg, 0.87 mmol) was added and the reaction mixture was stirred at room temperature for 3 mins. The green reaction mixture was then poured into deionized water (300 mL) and extracted with DCM (30 mL portions) until colorless. The organic layer was washed with saturated aqueous NaHCO_3_ solution (100 mL) and then water (100 mL), dried over Mg_2_SO_4_ and the solvent was removed under reduced pressure. The residue was redissolved in the minimum amount of DCM and purified on a plug of silica (eluent = DCM). The solvent was removed under reduced pressure and the solid remaining was dissolved in conc. HCl (60 mL). While stirring, tin(II) chloride (660 mg, 2.925 mmol) was added carefully. The final mixture was heated to 65 ˚C for 1 hr under Ar_(g)_. The green reaction mixture was poured into cold water (300 mL) and neutralized with ammonium hydroxide (35 %) to pH 10. The red product was extracted with DCM until the remaining layer was colorless. The organic layer was concentrated under reduced pressure and the residue was purified via column chromatography on aluminum oxide (eluent hexane : DCM, 2:1, v/v). The first fraction (purple) was found to be leftover TPP starting material. The second fraction (red) was found to be the product. The solvent from the second fraction was removed under reduced pressure and the product was recrystallized from DCM/MeOH. Purple crystals (86 mg, 0.136 mmol, 28 %) were obtained and gave analytical data consistent with the literature.^1^ ^1^H NMR (CDCl_3_): *δ* = 8.94 (d, *J* = 4.8 Hz, 2H), 8.83 (s, 6H), 8.22 (d, *J* = 7.7 Hz, 6H), 8.00 (d, *J* = 8.1 Hz, 2H), 7.82 – 7.63 (m, 9H), 7.07 (d, *J* = 8.2 Hz, 2H), 4.03 (s, 2H), -2.75 (s, 2H) ppm; HRMS (MALDI-TOF) [C_44_H_31_N_5_] [M]^+^: m/z calcd. 629.2579; found 629.2576. UV-vis (CH_2_Cl_2_) λ_max_: 419 (ε 680,450), 516 (ε 34,716), 554 (ε 22,092), 592 (ε 16,411) and 649 nm (ε 12,203).


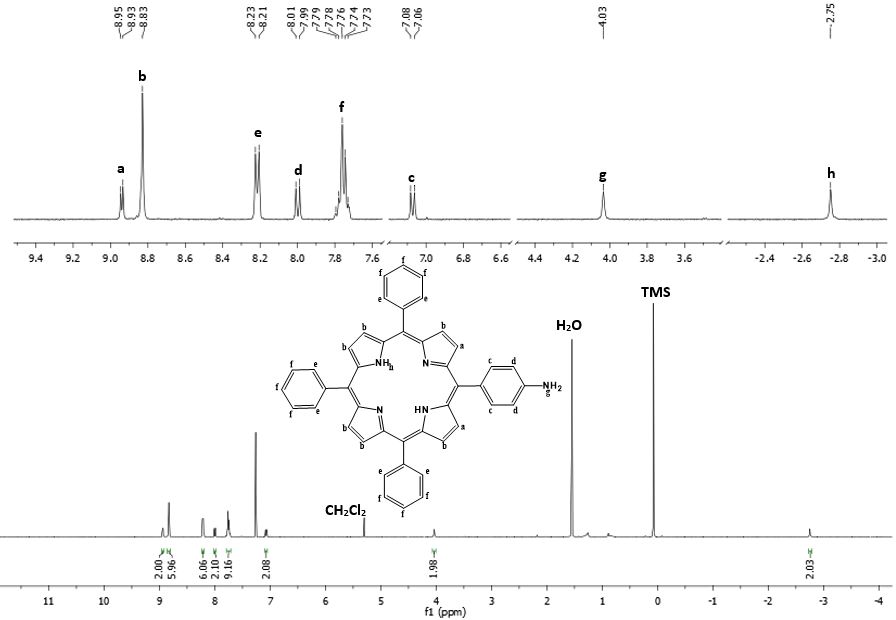


# Figure S1 ^1^H NMR spectrum of 5-(4-aminophenyl)-10,15,20-triphenylporphyrin (400 MHz, CDCl_3_, 25 °C).


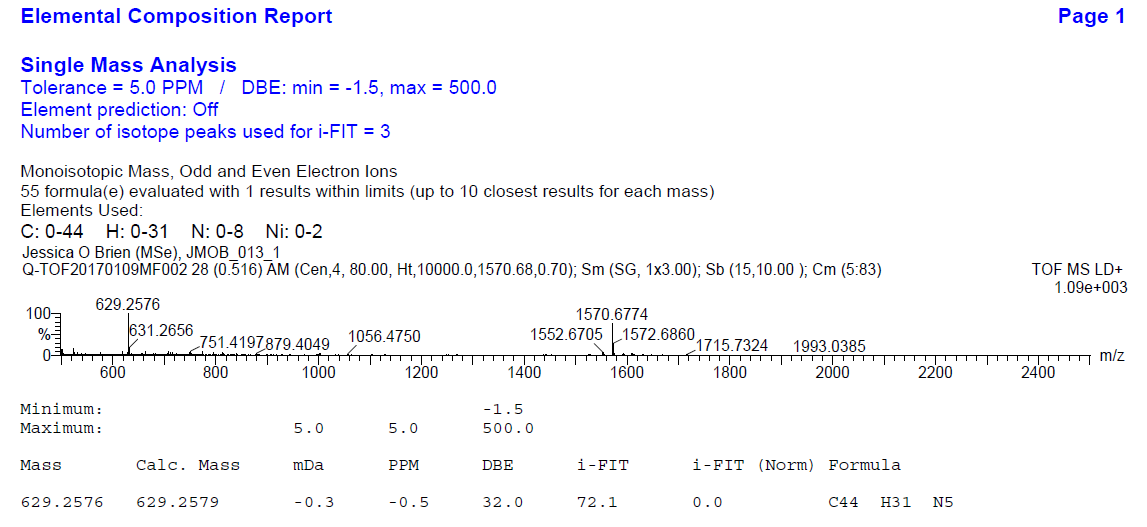


**Figure S2.** MALDI-TOF HRMS spectrum of 5-(4-aminophenyl)-10,15,20-triphenylporphyrin.

# Figure S3. UV-vis absorption spectrum of 5-(4-aminophenyl)-10,15,20-triphenylporphyrin in CH_2_Cl_2_.

**Figure S4.** The FTIR spectra of the GO, TPPNH_2,_ and the GO-CONHTPP nanohybrid. Red stars denote peaks attributed to TPPNH_2_.

Figure S5. Baseline corrected Raman spectra of the GO, TPPNH_2,_ and the GO-CONHTPP hybrid excited at 532 nm. Asterisks denote substrate signals (Si peak at 519 cm^−1^).

**
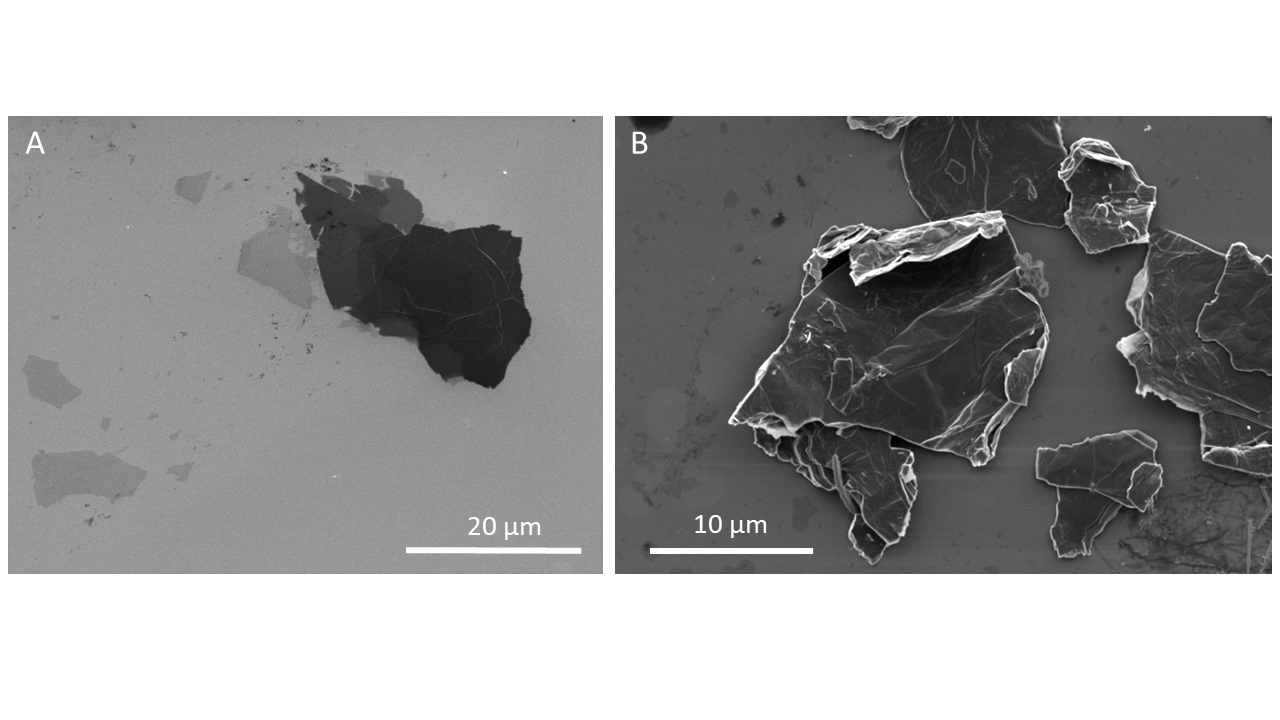
**

**Figure S6.** SEM images of the A) GO and B) GO-CONHTPP.

# Figure S7 UV-Vis absorption spectra of solution of TPPNH_2_ in DMF (black line) and in DMF-H_2_O (1:2 v/v) (red line).

**Figure S8.** Absorption spectra recorded during addition of A) DMF solution of TPPNH_2_ (0- 3.5 µM) to 0.13 mg mL^-1^ GO in DMF (3 mL), B) DMF-H_2_O (1:2, v/v) solution of TPPNH_2_ (0- 5.2 µM) to 0.26 mg mL^-1^ GO in DMF-H_2_O (1:2 v/v) (3 mL). Inset: absorption spectra of the TPPNH_2_ in its free form and adsorbed onto the GO surface obtained after subtracting the GO absorbance.

#

# Figure S9. Absorption spectra of GO suspension (black line), with the addition of TPPNH_2_ (red line), spectrum of the supernatant after centrifuging (blue line), suspended precipitate after centrifugation (dashed green line).





**Figure S10.** Molecular structure and ionic equilibria of the TPPNH_2_ in DMF-H_2_O (1:2 v/v).

# Figure S11. Absorption spectra recorded during addition of 1 M HCl to solution of TPPNH_2_ in DMF-H_2_O (1:2, v/v) and fitting of Boltzmann function to the relationship between absorbance and pH value, A) pH range from 7.37 to 2.96 A) pH range from 2.74 to 1.5.

# Figure S12. Absorption spectra recorded during addition of 1 M HCl to solution of GO-CONHTPP in DMF-H_2_O (1:2, v/v) and fitting of Boltzmann function to the relationship between absorbance and pH value.

# Figure S13. Normalized fluorescence excitation spectrum (λ_exc_= 670 nm) of TPPNH_2_ (red) and GO-CONHTPP (black) in DMF.

**Figure S14.** Measured quenching of the ﬂuorescence of TPPNH_2_ in DMF recorded during addition of a suspension of 2 mg mL^−1^ GO (0-0.067 mg mL^−1^), λ_exc_ = 419 nm with no I and II inner filter corrections.

**Figure S15.** Normalized fluorescence excitation spectrum (λ_exc_= 670 nm) of the mixture of TPPNH_2_ and GO (black), absorption spectrum of this mixture (blue) and normalized absorption spectrum of TPPNH_2_ (in the absence of GO (red) in DMF-H_2_O (1:2, v/v).

# Figure S16. Decay of TPPNH_2_ fluorescence in DMF-H_2_O (1:2 v/v) recorded in porphyrin concentration 0.5 μM (red) and 4 μM (black); *λ*_ex_ = 405 nm, *λ*_em_ = 650 nm.

# Figure S17. Decay of TPPNH_2_ fluorescence in DMF-H_2_O (1:2 v/v) recorded for porphyrin (black) and porphyrin with the addition of GO (0.013 mg ml^−1^) (red) ; *λ*_ex_ = 405 nm, *λ*_em_ = 650 nm.

**Figure S18** Transient absorption kinetics measured for the TPPNH_2_ in DMF-H_2_O (1:2 v/v) following the 422 nm laser excitation at A) 452 nm and B) 520 nm (black line shows the three exponential decay fit).

# Figure S19 Transient absorption spectra registered at various time delays for GO (0.4 mg mL^−1^) in water following 422 nm laser excitation.

# Figure S20. Decay curves of the singlet oxygen generated by free TPPNH_2_ (black), GO-CONHTPP (red) in DMF-H_2_O (1:2, v/v) (λ_exc_ = 408 nm, λ_mon_= 1270 nm, collection time: 30 min).

**Cartesian coordinates of optimized structures**

**TPPNH_2_**

C -0.12925261685121 -1.32856752706585 -2.60459857011076

C -0.13552916358275 2.13696619852400 0.84135395385323

C -0.19273203381286 -1.32679876495901 4.34395829280196

C -0.17814339294941 -4.79218289227218 0.89163331401650

N -0.12998954254619 0.17189680136186 2.36183460226514

N -0.14963610010661 0.10184936317654 -0.57772463012355

N -0.13850131545314 -2.82675841600133 -0.62541280820895

N -0.20538257976938 -2.75712661589195 2.31286916035709

C -0.22479523972103 -4.17852179675871 -0.37022153710791

C -0.20125770783462 -2.58443953344373 -1.98048027953328

C -0.05824540289652 -0.08911034144323 -1.93385665680950

C -0.05961032928489 1.45961159108972 -0.39335872975233

C -0.11609295026557 -4.11450455852785 2.12841325453568

C -0.11797817764128 -2.56622982202791 3.66989803035622

C -0.24294608696137 -0.07043479451259 3.71409576996305

C -0.21080722802714 1.52273929724002 2.10305724009240

C -0.37094857656592 -4.82750796297893 -1.64212725719496

C -0.35655827548346 -3.86038229451610 -2.61922228341941

C 0.13362821241974 1.18131949530573 -2.62686779442388

C 0.13291962383328 2.14584613080524 -1.66749227252570

C -0.40208671255404 2.17233097718092 3.36799824710212

C -0.42465604748146 1.20488824789844 4.34529072914317

C 0.07061868360233 -4.80161267806693 3.40218639313489

C 0.07221379571799 -3.83683802350614 4.36184509090898

H -0.49073518789992 -5.89729803216907 -1.77468783189808

H -0.46217851691714 -4.00186890949607 -3.68939069179840

H 0.27705373869446 1.30587770031776 -3.69583340498783

H 0.27636220838374 3.21410277269321 -1.79776588751442

H -0.53116745944574 3.24173256897026 3.49491231764933

H -0.57730741810029 1.34521311609399 5.40973781512572

H 0.21428325501706 -5.86994243782430 3.53192524693931

H 0.21961159043325 -3.96032554340417 5.43021902952912

C -0.13341422587275 -1.32229902217343 -4.09408577530532

C 0.90666193654176 -1.92641338767707 -4.81883475326704

C -1.18120405844272 -0.71127603009957 -4.80193412951167

H 1.72956954774421 -2.39213624453189 -4.27492339528480

H -1.99497359457299 -0.24682028859982 -4.24378177684807

C 0.90106927503100 -1.91758608941655 -6.21477320495101

C -1.18931005552231 -0.70553876050020 -6.19767455258390

H 1.72250246028797 -2.38267808681423 -6.76202739594391

H -2.01495383223450 -0.23268582000256 -6.73176994096493

C -0.14780176240497 -1.30808699625515 -6.90872865969092

C -0.14848692295042 3.62627256273332 0.82542563577416

C -1.19439409910168 4.32451910091899 0.20015037691250

C 0.88162140544921 4.36054973048218 1.43519619794187

H -2.00090577474747 3.75888987468353 -0.26782870341323

H 1.70256733756595 3.82402800561436 1.91278366725798

C -1.20989452150737 5.72011686406089 0.18520321788132

C 0.86890535442690 5.75629976430257 1.41670543348459

H -2.03400875069550 6.24672980124868 -0.29859729924270

H 1.68297697877481 6.31100940626038 1.88587626243282

C -0.17769075791253 6.44062234557263 0.79246899203094

C -0.22623741754566 -1.33363857341542 5.82835908678339

C -1.24685601401628 -2.00117819127651 6.52705698169041

C 0.76005054481608 -0.67866549161787 6.58531488562629

H -2.02938820187077 -2.50829884423817 5.96140710923726

H 1.57315650218680 -0.16832379001622 6.06719450342615

C -1.28574949349483 -2.01418861896053 7.91732753874808

C 0.73615699343872 -0.69228220869835 7.97628302978264

H -2.10185032400654 -2.52541160765758 8.43308268003248

H 1.52505225113871 -0.18702589048266 8.53800951123762

C -0.29052543897750 -1.36131165740164 8.66912958479562

C -0.19711335904962 -6.28139044276466 0.90994927782679

C 0.83974277538979 -7.02204461352397 0.31966303463660

C -1.25577691046408 -6.97323454923274 1.52071141259696

H 1.67144687527953 -6.49088362444631 -0.14515067819251

H -2.06661027459528 -6.40272542721217 1.97511607423858

C 0.82028987757802 -8.41769098981465 0.34161659139292

C -1.27789987413757 -8.36867043893568 1.53917948245811

H 1.63943111611400 -8.97728137271028 -0.11268385734213

H -2.11201113656374 -8.89019139560469 2.01124499688846

C -0.23947724864572 -9.09559032182906 0.95039421665960

H -0.06310888246468 -0.55479520240873 1.64678581681806

H -0.09949905772275 -2.09975617178682 0.09156667991994

N -0.35476569802288 -1.32669671338587 10.05995475917146

H 0.52165117028018 -1.14061251985507 10.53748674032996

H -0.88555930857980 -2.07495955550726 10.49531234679982

H -0.15302659911865 -1.30181802325696 -7.99995897631312

H -0.18844677664504 7.53175315330029 0.77862796938828

H -0.25549430510688 -10.18660763985909 0.96684083828860

**Covalent hybrid**

C 11.46701737633054 -17.78750155877762 2.44019816056257

C 11.04249326087969 -17.11811545535723 1.19145393866758

C 11.46648688198483 -19.14072891869968 2.51021605627121

C 10.40665419260737 -17.95436692153746 0.08767754093426

C 11.02252482150883 -19.97606072862219 1.42051051773170

C 10.52428882709915 -19.45850091759009 0.26927959719039

C 12.28870030229796 -15.51673817632475 3.27196758578862

C 12.80967589533680 -15.41305761798570 1.84415522849181

C 11.79391227986204 -16.93961334261020 3.63741150586455

C 11.72630142192516 -15.85049072123452 0.87086032945120

C 13.59380004175696 -13.36876364708441 4.06059066243122

C 13.97151302288834 -13.18275095196508 2.60183311946275

C 12.74561161808806 -14.59507513923919 4.34895994209570

C 13.46520219409998 -14.07601197756902 1.51843250284007

C 15.44160430351906 -11.80898277327241 4.62386822560753

C 15.12025388820824 -11.06869978220600 3.36957580342295

C 14.80587001377560 -13.01293530921060 4.88393892124748

C 14.37250826215664 -11.76030147380432 2.30363332235654

C 17.13853062239309 -10.13746767931675 5.17659386498496

C 16.94673045693836 -9.51169108375078 3.88947150923343

C 16.42222622244125 -11.26267424641010 5.50154780524545

C 16.01697877561560 -9.95709783584611 3.01154632872394

C 15.92435982487004 -9.37996856548579 1.63897432032785

C 15.29566476794550 -10.17689222506104 0.51377509221396

C 14.72122397988431 -11.54637375379292 0.85174214422612

C 13.57318210483026 -12.02996507462878 -0.00360892660464

C 13.17641519999891 -13.46104837051017 0.19548436450830

C 12.39551325105029 -14.08364463416051 -0.74477124811266

C 11.79355409504936 -15.37947452103030 -0.53302676437011

C 11.22184793164800 -16.13666276915710 -1.53783724100766

C 10.74342412153566 -17.48037196027151 -1.32061241517311

C 10.46735053023143 -18.34108702421149 -2.35916437922406

C 9.97992278632406 -20.22602847632867 -0.83678280550418

C 9.92911753666319 -19.63840949391839 -2.07560889107801

C 11.07042575272503 -15.58500248828996 -2.90634077497980

C 10.88815849426212 -16.50319572626409 -4.07370223112161

C 10.64842971867292 -17.99067887892561 -3.82009474050695

C 12.11763509921359 -13.40279653905655 -2.03533504858403

C 11.51058903511865 -14.18110513362307 -3.12698495669318

C 13.32068683733959 -11.36416078361191 -1.28433067555956

C 13.00972678983940 -12.25097676401056 -2.45589956416431

C 14.86191239662871 -9.52185435058291 -0.73233944190176

C 13.99912178565789 -10.08521917974579 -1.60766217952820

C 11.45918258243738 -16.12541831729403 -5.38344981257500

C 11.99492606425930 -17.08595776998022 -6.17743720623594

C 11.57151476193915 -18.89355232047905 -4.61485624725215

C 12.09905339325465 -18.46942737306432 -5.78655423019613

C 11.89527518931812 -13.72336880033126 -4.50743455453769

C 11.83970268338614 -14.70653774237929 -5.62797322499143

C 12.51218226883237 -11.52360266083134 -3.68812579349352

C 11.93495115961506 -12.25411924780939 -4.67379727153746

C 13.53951734667811 -9.44805490721078 -2.83184568405343

C 12.78057041711595 -10.10061676963577 -3.77570056258793

C 10.59628619732035 -16.86113547513814 4.59802177334635

O 10.68105740482190 -16.54829879444143 5.77850886151869

O 9.42579190607127 -17.19506600712649 4.03611417192959

C 9.47496668984939 -21.62301805067665 -0.77371371383825

O 9.10858900260342 -22.27201008805821 -1.73894646354198

O 9.41831224585968 -22.14816910958874 0.49148554715316

O 11.85379752708110 -19.74068666181991 3.68375443535688

O 12.77378049311511 -14.93350984672719 5.66882976209729

O 15.19009305654511 -13.86220572961738 5.84319231991621

O 10.36518483207867 -15.83945964574149 1.40516068121076

O 11.44610055627142 -14.42681341368249 3.70911256085388

O 14.87577704121078 -14.10509951258086 1.96683715696241

C 18.12424252362796 -9.53544991523191 6.09007303897861

O 18.79099964935694 -8.54318725656547 5.84281519302189

O 18.24063114328377 -10.20641730333961 7.28227424870302

C 15.90432474637650 -7.86957619282081 1.50415016871705

O 16.37340873017087 -7.24171491620124 0.57588843486653

O 15.24131125495979 -7.29647617771346 2.53775678271795

C 12.29095272934886 -9.37129962065592 -5.00662558408093

O 12.57936402941482 -9.73842411333460 -6.14354249448227

C 11.60569014011089 -14.17835058775416 -7.02573487838346

O 10.59124849272112 -14.39979239413489 -7.65360261502551

O 12.63161674126147 -13.41765356861495 -7.47629495481871

O 11.69011943319059 -20.15540608037221 -4.12518724952841

O 9.76769217806064 -15.75854510520666 -3.55125310788699

O 13.10819569714085 -14.39320875125389 -4.99127999113596

O 10.71567114642164 -13.25393923293646 -2.35586962484437

O 13.86909138158813 -8.14067860589748 -3.05587385542487

O 12.37650225286768 -11.19658429498067 -0.19147969438582

O 16.74162642519455 -10.06507201939245 0.67517679381284

O 13.67758281569025 -10.74687135048021 3.03270979286468

H 9.31490768313489 -17.75251531169208 0.18170548801592

H 11.02440721658867 -21.05608005486301 1.56886145234705

H 13.62333455706299 -16.15504531925900 1.74537717386732

H 12.58822188663065 -17.42668689518030 4.22730454882339

H 12.89329475008812 -12.53508662883232 4.24519981182325

H 17.59326186130305 -8.66719078108569 3.64707501500993

H 16.58253090180781 -11.70307205540062 6.48860490202732

H 15.53433928361437 -12.26226661717220 0.64444873432593

H 9.52248018051609 -20.23350943389612 -2.89254257417910

H 9.65442990417411 -18.16495810397270 -4.29089133447950

H 13.98597567943554 -12.70253090850800 -2.72689623480945

H 15.38562662870664 -8.58751512369188 -0.94828175005968

H 12.48128588243650 -16.78912390234168 -7.10876053454262

H 12.66288621586381 -19.15831759267290 -6.41930075028518

H 11.64077114925987 -11.80094842463820 -5.61593209498248

H 8.74327035769682 -17.07421460763623 4.73045055730587

H 9.05295197909719 -23.04911186289697 0.36712718740075

H 11.80295388797958 -20.70622759751100 3.57392407444065

H 12.03087388140672 -15.57498261313344 5.85983322473115

H 16.03157574813827 -13.54204407190081 6.22668322889667

H 18.91970614118332 -9.71334787194867 7.78600713159245

H 15.23877952802197 -6.33239478626777 2.35751841177256

H 12.34661821302150 -13.06409127902038 -8.34548952074398

H 12.22151927946378 -20.68701704074665 -4.74720840965013

H 14.27581446173629 -7.77400179078800 -2.24814834275855

C 4.49381889946391 -0.75760892644543 -12.11683547945634

C 9.23893348238051 -1.91443761588817 -11.94244867494309

C 8.39433397050380 -4.86564742892185 -8.09217327092426

C 3.64976520080670 -3.69798506473360 -8.25937877660847

N 8.49369219000753 -3.32450714143561 -10.03766406490612

N 6.79350108111005 -1.58241171384322 -11.68678954590172

N 4.38858358335488 -2.31893382557171 -10.18888788558448

N 6.10105908764437 -4.00726799121249 -8.49461212202620

C 3.45064961500081 -2.74812739949623 -9.27498619562801

C 3.83762550109181 -1.39553625693012 -11.05048818897500

C 5.86393941266946 -0.89522830514131 -12.42669608826510

C 7.98436288021705 -1.41140055026038 -12.34736140915354

C 4.88041745541044 -4.31288513147877 -7.94592300717525

C 6.99908718208659 -4.83797175235110 -7.87254861644604

C 9.07219410898789 -4.12301206044916 -9.07483075839550

C 9.45890625411180 -2.76716862322135 -10.84812464845611

C 2.23870516914160 -2.03671506202213 -9.56505629549150

C 2.47358650812048 -1.21529011422683 -10.64232379703997

C 6.47673118136730 -0.30290814762439 -13.61219545979209

C 7.79811067020187 -0.62336089815440 -13.56199912203167

C 10.72538065481630 -3.22603216671894 -10.35385348122199

C 10.49078186940408 -4.04727498283854 -9.27644414835855

C 5.00210055482286 -5.39574481095715 -6.97506780644628

C 6.32130243910558 -5.72520060390793 -6.93176842846546

H 1.31874327323546 -2.13965650633647 -9.00084278616547

H 1.77943019646002 -0.52718302856492 -11.11183022424897

H 5.95570107591380 0.25577647444910 -14.38322672072502

H 8.57170193820373 -0.37543355875622 -14.28161076877854

H 11.68509881111168 -2.93897690851066 -10.76832258113992

H 11.22578351057530 -4.54742867404392 -8.65603088974430

H 4.18251220398608 -5.85529992128207 -6.43226496900152

H 6.79107677837865 -6.51064891856442 -6.34868860080776

C 3.66455535459418 0.13581251259334 -12.97177469390700

C 2.57433341746553 -0.37123166061724 -13.69755032677115

C 3.95702106519816 1.50632681338470 -13.06723645019798

H 2.34828893634093 -1.43674031175684 -13.63832838125046

H 4.79736084462327 1.90820052462634 -12.50007026299843

C 1.79853515810869 0.46860104938784 -14.49816556951662

C 3.18079427432396 2.34643613914855 -13.86654184948503

H 0.96035589969510 0.05568067702882 -15.06151785748819

H 3.41894306275315 3.40977684493971 -13.92425215777896

C 2.09887815372167 1.83058059622535 -14.58507402329318

C 10.44070296975247 -1.51520472477495 -12.72695081093249

C 10.83773531390948 -0.16956589361865 -12.78876102592181

C 11.19924338283838 -2.47395161091761 -13.41754961095834

H 10.25665376956746 0.57875732140544 -12.24844473251637

H 10.88934365164801 -3.51943957635127 -13.38308565028072

C 11.96509903038163 0.20669029049236 -13.52049671706439

C 12.32429811708670 -2.09750659955113 -14.15286371356543

H 12.26497010304253 1.25532873338766 -13.55121518786152

H 12.89743126667262 -2.85409813307438 -14.69053708079452

C 12.71175666746078 -0.75586976648836 -14.20504934021039

C 9.22121088171746 -5.75134799357824 -7.23113923527492

C 9.24166251163106 -5.59114662906592 -5.83499907422977

C 9.99501031040747 -6.78443556078027 -7.78319851533639

H 8.66153435166908 -4.78670940227611 -5.38209955261144

H 9.98346624271344 -6.93240160168846 -8.86416010131501

C 9.99366165307283 -6.43767590099405 -5.02946574480411

C 10.75523323882678 -7.64186603768289 -6.99119915787542

H 9.99560115792911 -6.29534443178503 -3.94565186604665

H 11.34025114043258 -8.44273676919129 -7.43276681292673

C 10.75327681118424 -7.47676364371113 -5.59562153464100

C 2.45385134011834 -4.07309348814855 -7.45481343161343

C 1.34910144195459 -4.69828868357329 -8.05592187272486

C 2.40935325713726 -3.80611456438236 -6.07683912054279

H 1.38231592331201 -4.92125385618588 -9.12321543964641

H 3.26214680086498 -3.31466038398700 -5.60632676433268

C 0.23026358957906 -5.04911570043632 -7.29860666715028

C 1.28895967981393 -4.15258830828703 -5.32012962366143

H -0.61519818171706 -5.54394379701050 -7.77916520728356

H 1.26758770984328 -3.92996701960939 -4.25232395752073

C 0.19596931336887 -4.77590335275442 -5.92862533618301

H 7.49400290603137 -3.11976697053215 -10.09764534439481

H 5.36921729015435 -2.60649315115172 -10.19690790030143

N 11.45506206232576 -8.31848862198497 -4.71811698539462

H 11.34443409146658 -8.11129910550870 -3.72865415206220

H -0.67854694974899 -5.04965972955219 -5.33684482066533

H 13.59303775578512 -0.46135735564275 -14.77635416937989

H 1.49224790722215 2.48690635371478 -15.21060154182975

**Non-covalent hybrid**

C 12.89289914033276 -18.60453807987074 1.00657601758702

C 11.98192696789486 -17.60416798110767 0.41667744577063

C 12.79038894191806 -19.90444517200646 0.62991822629646

C 10.92419765429208 -18.07555853316738 -0.58688484503289

C 11.79391696564589 -20.39125814454457 -0.28695616208111

C 10.93039020291892 -19.55333611071411 -0.90764913649581

C 13.95126688791888 -16.69291766031775 2.31939752432085

C 13.78708064021047 -15.87574562465726 1.03232505922510

C 13.94703756070550 -18.21187507274707 2.01720869977129

C 12.44402999363641 -16.19839143861659 0.38444131624216

C 14.85801713699270 -14.61517669961390 3.68503680638360

C 14.69610431578510 -13.80581740740040 2.40727850790091

C 14.59560645284506 -16.10903423722244 3.53925380206995

C 14.04821169003118 -14.37792863414510 1.18642730318393

C 16.14506740815017 -12.73544294951620 4.67482610456476

C 15.32370062035651 -11.80451880939445 3.85837844507126

C 16.06623000141715 -14.09645429629909 4.43310633297908

C 14.61320955090041 -12.32165945970832 2.67333666787775

C 17.15777616963529 -10.84068320995377 5.83789144486392

C 16.52772196688408 -9.93737238990297 4.90648562366421

C 16.99948628860540 -12.19544270760603 5.67964760544796

C 15.69382146095355 -10.38225318255879 3.93730225532431

C 15.17650348859857 -9.45412642916890 2.89850879377583

C 14.58917979487995 -9.98898907188008 1.61654765677871

C 14.50103514862897 -11.49835601965368 1.41605281773698

C 13.32043087594489 -11.98995949313698 0.60987324553948

C 13.26354528733731 -13.45804907974985 0.32091770947944

C 12.32897253848720 -13.90660049638549 -0.57958515205339

C 11.96073622654914 -15.29750142065177 -0.69862735754563

C 11.15181452455606 -15.79643527223950 -1.70618209841424

C 10.80000249596916 -17.18966360406907 -1.81250723173355

C 10.19479589035196 -17.71644694852212 -2.93316820952903

C 9.96220610785851 -19.94900717000941 -1.91418610225689

C 9.64410723788636 -19.03628487812831 -2.88429439765337

C 10.58654028455521 -14.89204487508654 -2.73547423042304

C 10.12974499385848 -15.43785067909864 -4.05090288467590

C 9.98341306733804 -16.94521462909967 -4.21604293186419

C 11.63541818676327 -12.92461933465425 -1.45948558159557

C 10.86161955944877 -13.44073427459623 -2.59553986657348

C 12.64289259833385 -11.03304313993012 -0.27895835745296

C 12.23755436409325 -11.54007983781756 -1.62992658933369

C 13.75603449093991 -9.11414235077912 0.77817401696848

C 12.92045265684049 -9.58775805995184 -0.16888731989599

C 10.35208330112470 -14.63724860020488 -5.27189763712608

C 10.71376362351295 -15.26830910726727 -6.41719239236403

C 10.68761421468092 -17.49140915802227 -5.44000365586536

C 10.92640100615716 -16.69263705295182 -6.50674931517569

C 10.86348410720554 -12.55720581358423 -3.80741100556068

C 10.60073017961969 -13.17458438845364 -5.13853798023361

C 11.38272347601815 -10.58854977765950 -2.45983177288046

C 10.72324720732083 -11.11821379461800 -3.52063808767983

C 12.11308261076809 -8.70567044616851 -1.00051286914846

C 11.37949305203130 -9.16699903821961 -2.10017057495987

C 13.81215689723311 -19.05877681903978 3.29299952159863

O 14.76325249181957 -19.33002953251769 4.01957406070533

O 12.57140994381007 -19.45754615595948 3.56907900035951

C 9.31000954629299 -21.27565795999218 -2.02499747207903

O 8.56464777467357 -21.61246686789920 -2.93064654333123

O 9.58568876599643 -22.11912980403425 -0.98054682365320

O 13.69783920428206 -20.79954829536842 1.14394110789703

O 15.29362775847198 -16.81007986833008 4.47437873880308

O 16.97883850646212 -14.95806450908464 4.89587227925262

O 11.42542094929004 -16.60516181155493 1.33031775526242

O 13.15304968135228 -16.29733141753975 3.44921212079231

O 15.50426602045688 -14.11766841897812 1.26317575993053

C 17.95281963228311 -10.25894511524819 6.93080141542653

O 18.10815313989785 -9.06280273810182 7.12240767339204

O 18.52213098471084 -11.20159065064493 7.75153386760534

C 14.73503795636622 -8.07865445547011 3.36653668835708

O 15.00077695354155 -7.03006436991011 2.82144210165833

O 13.97414334927175 -8.18776923893118 4.48385534470600

C 10.57692464242097 -8.21973963103488 -2.90544557182312

O 10.01248866532433 -8.44717641310266 -3.96382551957235

O 10.45503084941309 -6.95357938791920 -2.34384715022061

C 9.99894441875264 -12.29283799187619 -6.20885056330030

O 8.83731625078531 -12.37247071714743 -6.55225510242053

O 10.88124132359779 -11.39796588139265 -6.71593869992169

O 10.90424355041424 -18.83214314031781 -5.40536946357571

O 9.16122753276034 -15.00358960080243 -3.07196061433927

O 11.96422769157949 -12.89832091465310 -4.72258764719843

O 10.19109687940621 -12.93870705839834 -1.41599106354438

O 12.14403337219339 -7.43257724458366 -0.59869852918591

O 11.97102186264819 -11.51766126941650 0.91345749667053

O 15.95371184495284 -9.46907682214700 1.69105999772431

O 13.82003304673440 -11.97382542830733 3.80888357646774

H 9.96431831385183 -17.91377506696510 -0.04116592121949

H 11.74759831741643 -21.46365908630089 -0.48298305981343

H 14.56822648614940 -16.22179817738177 0.33079834114692

H 14.94957714981323 -18.46724009366588 1.63034250963152

H 14.00750196519302 -14.27169652565147 4.30228528837738

H 16.75882965193116 -8.87597439098147 5.00730382256268

H 17.48412881063424 -12.85817803939167 6.40065238970218

H 15.38248618114939 -11.76752244101248 0.80915509613536

H 8.96581152114525 -19.35857977359306 -3.67314543905185

H 8.90644812283482 -17.06590055105626 -4.46857108272416

H 13.18575925245607 -11.67566036836955 -2.18728798057668

H 13.90560895161933 -8.04178962561647 0.89609083017315

H 10.96736967645473 -14.66677710470473 -7.29263108756082

H 11.32945943780402 -17.11140916912932 -7.43141413300273

H 10.16022373112176 -10.48431481970872 -4.19629325183003

H 12.59927944357603 -19.83430281754417 4.48050466894041

H 9.06913876064672 -22.92884626805826 -1.17478550962923

H 13.45702652570990 -21.69632963178882 0.85406417021303

H 15.27713083967494 -17.78654088152329 4.29183527193806

H 17.72097988975187 -14.43924648964562 5.26882489238051

H 19.01036302113510 -10.68738940606950 8.42616117971039

H 13.71563187666602 -7.27932739030310 4.74522202263745

H 9.47834602548487 -6.67185200145967 -2.46125841317624

H 10.36738497191000 -10.84045231473694 -7.33823736017130

H 11.26673844920111 -19.11696423124855 -6.26520323699402

H 11.47374856799567 -6.93973069606610 -1.16626388160509

C 10.57115702014430 -16.63157318411892 5.19655544328032

C 7.82968274978241 -16.10030150674194 1.18722431385063

C 8.61112705816141 -11.23496395704152 1.33063008355128

C 11.32444920887572 -11.76318905047383 5.36532632177574

N 8.40058827457462 -13.70723855591376 1.53828023120120

N 9.20964717712753 -15.95168759322237 3.23902724005766

N 10.75885398955494 -14.15604804401970 5.01119622724954

N 9.86773907306447 -11.90641778100905 3.35914592083807

C 11.27402449254307 -13.11346178009570 5.75454099698587

C 10.93558964642241 -15.35634138459128 5.66460025334105

C 9.82679562152083 -16.89066539339658 4.02352003865264

C 8.59767736518760 -16.65331515271765 2.23277303049339

C 10.74706083259996 -11.24741945662830 4.18156837834399

C 9.54158802022623 -11.00941269932776 2.37258961523001

C 8.02639578941936 -12.48332983104308 1.02932563669474

C 7.68829310917174 -14.72270513531420 0.93849414150324

C 11.77619892455124 -13.68953313264177 6.96803211185142

C 11.57578475025237 -15.04963579459105 6.91109358577037

C 9.63123331376155 -18.22946173922391 3.47395456576659

C 8.86602289612098 -18.08280751181104 2.35988398449245

C 6.77315512493181 -14.10009190056758 0.02964944786948

C 6.96935523431638 -12.74126356015801 0.09374502873823

C 11.03722511072142 -9.91399705288847 3.66843215914404

C 10.29503459409027 -9.76740119552981 2.53874903932983

H 12.21132079605324 -13.12326466915621 7.78328404649476

H 11.82270277998760 -15.78191256822480 7.67142258988812

H 10.05796653104874 -19.14807231376318 3.85553078133410

H 8.54240943486174 -18.86140094851921 1.67618590163601

H 6.04401507963262 -14.63308620802792 -0.57004936121693

H 6.42077217848778 -11.97718629267438 -0.44319526791939

H 11.76976160201899 -9.22759535350929 4.07835416123763

H 10.31225462001354 -8.93136469745517 1.84729348622063

C 11.05394303689918 -17.79846408791171 5.97723388313476

C 12.42172020200874 -17.95353415011104 6.26723852705677

C 10.16606555150398 -18.80614991121308 6.39625487007056

H 13.12721507407625 -17.19491340994760 5.93203245723100

H 9.10047549637911 -18.68357922284539 6.19945008780352

C 12.89035262821654 -19.09871340156516 6.91528799793363

C 10.63275796665451 -19.95134780855867 7.04370722352020

H 13.95911228756477 -19.20908664143003 7.10325102952954

H 9.92620665457819 -20.72188130572435 7.35562654365679

C 11.99931990771467 -20.10871134206572 7.29653803072962

C 7.13794680807599 -17.03177985663063 0.25938644145464

C 6.29102676897366 -18.04640352155055 0.74253144562785

C 7.33575525790066 -16.93234658803857 -1.12865328932095

H 6.10864889648225 -18.11591203456865 1.81547473246545

H 7.99159690571644 -16.15842019429074 -1.52578794550998

C 5.68445949255514 -18.94772944872213 -0.13222755424972

C 6.72050636516320 -17.82973770589022 -2.00176958236273

H 5.03087391580460 -19.72716727314699 0.26325662986985

H 6.88546393362348 -17.73357122488125 -3.07650122888133

C 5.90013667832082 -18.84751999636325 -1.50999107564430

C 8.26003885680674 -10.10715208569825 0.43666437314048

C 7.95782510278145 -8.82267455113483 0.92851091006177

C 8.24876896086952 -10.28515449353411 -0.95878137982858

H 7.90471882492665 -8.66566045431262 2.00579164703919

H 8.51465198082656 -11.25549187290383 -1.37314660219993

C 7.73877117803116 -7.74888312352279 0.06794432517457

C 7.98066075876782 -9.23116109773595 -1.82244486594616

H 7.54521870205070 -6.75522627784623 0.47829521281215

H 8.01406628712029 -9.39501142193225 -2.89920292457669

C 7.77196542633895 -7.94205847461019 -1.31894438754091

C 12.05833375570566 -10.81916065229256 6.24764964142442

C 13.37640945849960 -11.06985333742724 6.66894057113688

C 11.44126719706467 -9.62952530742548 6.67610954850961

H 13.87723576243798 -11.97429680525779 6.32832042175237

H 10.41295093948632 -9.43172084338002 6.37272107841749

C 14.05613995868469 -10.16162258914764 7.47815292665953

C 12.12191104617232 -8.71921019441624 7.48518088782131

H 15.08258921846379 -10.37195743423663 7.77839088341067

H 11.61979629615021 -7.80732244911102 7.81309128962117

C 13.43493431157990 -8.97953901490067 7.88732568147213

H 9.07640812224678 -13.84718650923558 2.29055699638780

H 10.26061253794383 -14.03887114937984 4.12724831910794

N 7.75054726135742 -6.83196669667619 -2.21548230436596

H 7.36369178648844 -7.06907850095857 -3.12998214556117

H 7.26851410853935 -6.01746186613211 -1.83485669976656

H 13.97168867886437 -8.26704331417599 8.51547909169459

H 5.43130806262509 -19.55615283516217 -2.19353165415989

H 12.36762992251210 -21.00569298308490 7.79633507864231

**References**

1. K. Ladomenou, T. Lazarides, M. K. Panda, G. Charalambidis, D. Daphnomili, A. G. Coutsolelos, *Inorg. Chem.* **2012**, *51*, 10548-10556.
